# Developmental disruption of amygdala transcriptome and socioemotional behavior in rats exposed to valproic acid prenatally

**DOI:** 10.1186/s13229-017-0160-x

**Published:** 2017-08-01

**Authors:** Catherine E. Barrett, Thomas M. Hennessey, Katelyn M. Gordon, Steve J. Ryan, Morgan L. McNair, Kerry J. Ressler, Donald G. Rainnie

**Affiliations:** 1 Silvio O. Conte Center for Oxytocin and Social Cognition, Division of Behavioral Neuroscience and Psychiatric Disorders, Yerkes National Primate Research Center, Emory University, 954 Gatewood Rd, 30329 Atlanta, GA USA; 2 Department of Psychiatry and Behavioral Sciences, Emory University School of Medicine, 30329 Atlanta, GA USA; 3 Department of Psychiatry, McLean Hospital, Harvard Medical School, Belmont, MA 02478 USA

**Keywords:** Valproic acid, Autism, Social behavior, Basolateral amygdala, Protein kinase A, Transcriptomics, Proteomics, RNA sequencing

## Abstract

**Background:**

The amygdala controls socioemotional behavior and has consistently been implicated in the etiology of autism spectrum disorder (ASD). Precocious amygdala development is commonly reported in ASD youth with the degree of overgrowth positively correlated to the severity of ASD symptoms. Prenatal exposure to VPA leads to an ASD phenotype in both humans and rats and has become a commonly used tool to model the complexity of ASD symptoms in the laboratory. Here, we examined abnormalities in gene expression in the amygdala and socioemotional behavior across development in the valproic acid (VPA) rat model of ASD.

**Methods:**

Rat dams received oral gavage of VPA (500 mg/kg) or saline daily between E11 and 13. Socioemotional behavior was tracked across development in both sexes. RNA sequencing and proteomics were performed on amygdala samples from male rats across development.

**Results:**

Effects of VPA on time spent in social proximity and anxiety-like behavior were sex dependent, with social abnormalities presenting in males and heightened anxiety in females. Across time VPA stunted developmental and immune, but enhanced cellular death and disorder, pathways in the amygdala relative to saline controls. At postnatal day 10, gene pathways involved in nervous system and cellular development displayed predicted activations in prenatally exposed VPA amygdala samples. By juvenile age, however, transcriptomic and proteomic pathways displayed reductions in cellular growth and neural development. Alterations in immune pathways, calcium signaling, Rho GTPases, and protein kinase A signaling were also observed.

**Conclusions:**

As behavioral, developmental, and genomic alterations are similar to those reported in ASD, these results lend support to prenatal exposure to VPA as a useful tool for understanding how developmental insults to molecular pathways in the amygdala give rise to ASD-related syndromes.

**Electronic supplementary material:**

The online version of this article (doi:10.1186/s13229-017-0160-x) contains supplementary material, which is available to authorized users.

## Background

The etiology of autism spectrum disorder (ASD) likely involves complex interplay between genetic and environmental factors. As over 800 genes are implicated in the etiology of ASD (Simons SFARI database), any one knockout will not entirely represent the complexity of core abnormalities. Moreover, although genetic heritability plays an important role in the etiology of ASD, a number of early environmental exposures have also been linked to ASD risk [1]. The antiepileptic drug and mood stabilizer, valproic acid (VPA), has a well-documented history of increasing the susceptibility to ASD. Children exposed to VPA during the first trimester of pregnancy are at increased risk of developing ASD, with estimates varying between 2.9 and 18-fold greater risk compared to the general population [2–4].

Rats exposed to VPA prenatally around embryonic day (E) 12.5 display abnormalities in neurological and behavioral development. Notably, the VPA rat model recapitulates many of the core symptoms of ASD, including impaired social behaviors, increased repetitive behaviors, and communication impairments, as well as hyperserotonemia, heightened dopamine levels, increased ratio of excitatory to inhibitory neurotransmission, elevated physiological and behavioral measures of anxiety, and enhanced responsivity to sensory stimulation [5–9].

VPA alters neural circuits in brain regions implicated in ASD, such as the amygdala, and thus is a useful tool to investigate how the disruption of these circuits can lead to emotional and behavioral abnormalities. Abnormal functioning of the amygdala has long been implicated in the etiology of ASD [10]. Amygdala abnormalities in structure [11–13], neuronal density [14, 15], and functional MRI activity during social tasks [16–22] have been reported in patients with ASD. Prenatal VPA treatment induces hyper-excitability, enhanced long-term potentiation, and hyper-plasticity of neurons in the amygdala, a reduction in inhibitory synaptic transmission [23, 24], and local hyper-, but distal hypo-connectivity of neural microcircuits [25]. Local amygdala hyperactivity contributes to enhanced fear memories, over-generalized fear, reduced fear extinction, and enhanced anxiety in rats prenatally exposed to VPA [23, 26, 27]. The impact of VPA on local hyperactivity and hyperplasticity is in line with the “intense world” theory of ASD, which postulates that excessive neuronal processing leads to a hyper-functionality underlying ASD symptomatology [28]. Impairment in GABAergic circuitry [29] and under-connectivity between brain regions [30, 31] are also similarly implicated ASD etiology.

Premature amygdala development is commonly reported in youth with ASD [32–34], potentially contributing to the observation of clinically significant anxiety in 40% of children and adolescents with ASD, twice that seen in typically developing children [35–37]. The basolateral amygdala (BLA) is an important center for multimodal sensory information processing in the control of emotional arousal and social behavior [38]. We have previously shown that the rat amygdala undergoes phenomenal changes in the first few weeks of life in GABA and glutamatergic signaling, structure, and function [39–41]. At postnatal (P) day 10, the ability to learn fear association emerges, along with functional, molecular, and physiological changes in the amygdala [39, 42–44]. Between P7 and 28, extensive changes occur in molecular and electrophysiological properties of BLA principal neurons [39, 41] and in GABAergic fiber and cell body density [45]. Behaviorally, amygdala-mediated fear learning and extinction undergo dynamic changes between juvenile and adolescent stages [46]. Plasticity in BLA neurons is bidirectional and under very tight control by second messenger systems like cAMP-Protein Kinase A (PKA) [47, 48]. However, surprisingly little is known about the cellular, molecular, or genetic changes that occur in the amygdala over development in ASD individuals or rodent models. In order to develop appropriate early interventions and treatments for neurodevelopmental disorders, it is important to investigate changes across development. Appropriate treatments may differ greatly across time, depending on the natural biological state of neurons in the amygdala. Here, we investigated the gene expression in the BLA across development in prenatally VPA-exposed rats. This is the first comprehensive analysis of genetic changes in this region over development.

## Methods

### Animals

Pregnant Sprague-Dawley dams (Charles River, Wilmington, MA) arrived at our animal facilities at 5–6 days of gestation and were maintained on a 12:12 h light-dark schedule with access to food and water ad libitum. Day of birth was considered postnatal day (P) 0. Pups were weaned at P21 and group housed with same-sex, same-treatment siblings. No more than two animals of the same sex and litter were used per behavioral experiment. No more than one animal per sex and litter were used in genomic analyses. A timeline of experiments and cohorts can be found in Table 1. All protocols strictly conformed to the Guidelines for the Care and Use of Laboratory Animals of the National Institutes of Health and were approved by the Emory University Institutional Animal Care and Use Committee.

**Table 1:** Outline of experiments

Age	Measure	Cohort 1	Cohort 2	Cohort 3	Subjects
	*Behavior*				
P1–6	Maternal behavior		X		Dams
P7, 11	Ultrasonic vocalizations		X		Males, females
P9	Maternal approach	X			Males, females
P8–10	Fear conditioning			X	Males, females
P13–18	Odor-potentiated startle		X		Males
P14	Eye opening	X			Males, females
P32–35	Open field, social interaction	X			Males, females
P36	Startle	X			Males, females
	*Genomic analysis*				
P10	RNA seq	X			Males
P21	RNA seq, proteomics	X			Males

### Drug administration

As intraperitoneal VPA administration leads to high rates of fetal resorption [49, 50], an oral route of administration was chosen, which also mimics clinical usage. Pregnant females received oral gavage with either 500 mg/kg of VPA (Sigma-Aldrich, St. Louis, Missouri; ~800–900 ul) diluted in 0.9% saline or saline alone on embryonic (E) days 11–13. Offspring were weighed between P3 and 50 and assessed for eye opening on P14.

### Behavior

#### Maternal behavior

Home cage observations were conducted daily from P1 to 6 (16:00–17:00) on VPA (*n* = 6) and saline (*n* = 4) treated dams. Point observations of position of dams on the litter (on or off), nursing style (arched-back, blanket, passive), and licking/grooming pups were made every 4 min (15 obs/hour).

#### Ultrasonic vocalizations

On P7 and 11, pups (*n* = 10/grp) were removed from the home cage and individually placed into sound-proofed cages (31.7 × 17.2 × 14.2 cm) for 5 min. Ultrasonic vocalizations (USVs) were recorded and analyzed for the number, frequency, and duration using Sonotrack software (Metris, Netherlands).

#### Nest-seeking response

On P9, the latency to approach maternal bedding was assessed (*n* = 14–16/grp). Bedding from the maternal home cage and clean bedding was placed on filter paper at either corner of the testing cage (31.7 × 17.2 × 14.2 cm), and a live observer recorded latency to approach either bedding.

#### Infant fear learning

The impact of VPA on the development of fear learning was assessed by light-shock conditioning infant rats (P8–10; *n* = 20–23/grp). Heating pads were placed underneath the shock grid as young rats are not able to regulate body temperature. On P7, rats were habituated for 20 min to chambers and exposed to 30 noise bursts (95 dB). On P8–10, rats received 2–3 days of conditioning consisting of a 5-min acclimation, followed by 15 startle leaders (95 dB), and 10 light-shock pairing (1 s light terminating with 0.6 mA shock). Half of each group received 2 days and half received 3 days of conditioning, but data were combined for analysis as both paradigms yielded similar results. After 12 days (P21–22), the animals were returned to the original chambers for a 5-min acclimation, followed by exposure to 30 light-tone (95 dB) and 30-tone alone trials. Fear-potentiated startle was calculated by normalizing startle amplitudes to the acclimation period responses and calculating the percent increase from tone alone to light-tone trials.

#### Startle to maternal odor

In order to assess maternal attachment learning, we exposed pups to 10% acetophenone (Sigma) scented food from P0 to 10 to create a maternal odor as described by Todrank et al. [51]. Standard laboratory chow was mixed with 10% acetophenone (Sigma) in propylene glycol (1 ml per 100 g chow) and allowed to air dry in a fume hood for 3 days. Scented food was provided as the sole food source from P0 to 10, and then regular laboratory chow was provided. Acetophenone odor-potentiated startle (OPS) was assessed in male offspring (P17–20, *n* = 17 saline, 14 VPA) as previously described [52]. Testing consisted of 15 startle leaders (105 dB) followed by 10 odor-startle (10 s odor ending in 50 ms 105 dB noise burst) and 10 startle-alone trials randomly interspersed. A subset of the animals was also tested for OPS to a neutral odor (*n* = 10 saline, 8 VPA). The procedure was run on two consecutive days with the odor being acetophenone (maternal) or propanol (neutral) in a counter-balanced fashion. Percent OPS was calculated by subtracting the startle amplitude during the last startle leader from the first odor-startle trial, dividing by the last leader startle amplitude, and multiplying by 100.

#### Open field and social behavior testing

At P35, subjects (*n* = 22–26/grp) were habituated to the novel testing arena (27.3 × 90.2 × 91.1 cm) for 5 min under red light illumination, which also served as an open field test of basal anxiety levels. Subsequently, a cage containing a same-sex, same-age conspecific and an empty cage were placed at opposite corners of the arena to test the subject animal’s preference to spend time in proximity to another rat or an object. After 5 min, a novel same-sex, same-age conspecific was put into the empty cage, and the preference for a novel or familiar animal was assessed over 5 min. For analysis, the arena was divided into four quadrants, each approximately 45 × 45 cm. The social preference portion contained a social zone, an object zone, and two empty zones. Subsequently, the social novel portion contained a familiar zone, a novel zone, and two empty zones. Durations in and entry bouts into the zones were analyzed using an automated system (Cleversys, TopScan).

#### Acoustic startle

At P36, rats (*n* = 22–26/grp) were presented with 10 trials each of 95, 100, and 110 dB acoustic stimuli, along with 10 prestimulation trials with no noise burst. A fast-rise-time (<1 msec) burst of noise presented for 40 msec was used, with intertrial intervals of 30 s.

### Brain collection

Subjects were deeply anaesthetized with isofluorane, decapitated, and brains were frozen on dry ice and stored at −80 °C. BLA punches (~1μm^3^) were collected on a microtome.

### Next generation RNA sequencing

Transcriptomics was assessed in male amygdala micropunches from P10 (*n* = 4 saline, 4 VPA) and P21 (*n* = 4 saline, 3 VPA). RNA-sequencing libraries were prepared by the Yerkes Nonhuman Primate Genomics Core (NHPGC). Total RNA was prepared using the QIAGEN RNEasy Micro Kit. Libraries were generated from 5 ng of Total RNA using the CLONTECH SMARTer HV kit, and barcoding and sequencing primers were added using NexteraXT DNA kit. Libraries were validated by microelectrophoresis, quantified, pooled, and clustered on Illumina TruSeq v3 flowcell. The clustered flowcell was sequenced on an Illumina HiSeq 1000 in 100-base single-read reactions. Sequenced reads were processed using the Illumina BaseSpace Cloud environments RNAExpress App Workflow version 1.0.0. The reads were aligned to the UCSC (http://genome.ucsc.edu/index.html) rn5 reference assembly using the STAR Aligner [53]. Gene abundance estimation was done by counting the number of aligned reads that overlap annotated genes in the reference assembly using a custom script based on the method of htseq-count [54]. The per sample count files were loaded into the DESeq2 [55] R package for normalization and differential expression analysis. Library size normalization was performed and differential expression was calculated with a two-factor two-level crossed model using the negative binomial Wald test, and Benjamini-Hochberg False Discovery Rate (FDR) was use for multiple comparisons.

### Proteomics

Liquid chromatography–tandem mass spectrometry (LC-MS/MS) was performed by the Emory Integrated Proteomics Core using contralateral P21 amygdala micropunches from the same animals that were run for RNA sequencing (*n* = 4 saline, 3 VPA). Tissue samples were homogenized in lysis buffer (8 M urea, 100 mM NaHPO4, pH 8.5), including HALT protease and phosphatase inhibitor cocktail (Pierce), using a Bullet Blender (Next Advance). Supernatants were centrifuged, sonicated (Sonic Dismembrator, Fisher Scientific), and vortexed. Protein concentration was determined by the bicinchoninic acid (BCA) method. Protein homogenates (100 μg) were treated with 1 mM dithiothreitol (DTT), followed by 5 mM iodoacetimide (IAA), digested with 1:100 (*w*/*w*) lysyl endopeptidase (Wako), diluted with 50 mM NH4HCO3, and further digested overnight with 1:50 (*w*/*w*) trypsin (Promega). Resulting peptides were desalted with a Sep-Pak C18 column (Waters).

Peptides were resuspended in loading buffer (0.1% formic acid, 0.03% trifluoroacetic acid, 1% acetonitrile), separated on a self-packed C18 (1.9 um Dr. Maisch, Germany), fused silica column (25 cm × 75 uM internal diameter (ID); New Objective, Woburn, MA) by a Dionex Ultimate 3000 RSLCNano, and monitored on a Fusion mass spectrometer (ThermoFisher Scientific, San Jose, CA). Elution was performed over a 120-min gradient at a rate of 300 nl/min with buffer B ranging from 3 to 80% (buffer A 0.1% formic acid in water, buffer B 0.1% formic in acetonitrile). The mass spectrometer cycle was programmed to collect at the top speed for 3-s cycles. MS scans (400–1600 m/z range, 200,000 AGC, 50 ms maximum ion time) were collected at a resolution of 120,000 at m/z 200 in profile mode, and the HCD MS/MS spectra (2 m/z isolation width, 30% collision energy, 10,000 AGC target, 35 ms maximum ion time) were detected in the ion trap. Dynamic exclusion was set to exclude previous sequenced precursor ions for 20 s within a 10 ppm window. Precursor ions with +1 and +8 or higher charge states were excluded from sequencing.

RAW data was analyzed using MaxQuant v1.5.2.8 with Thermo Foundation 2.0. The search engine Andromeda, integrated into MaxQuant 1, was used to build and search a concatenated target-decoy Uniprot rat reference protein database (retrieved April 20, 2015; 29,370 target sequences), plus 245 contaminant proteins from the common repository of adventitious proteins (cRAP) built into MaxQuant. Quantitation of proteins was performed using summed peptide intensities given by MaxQuant. The full list of parameters used for MaxQuant is available as mqpar.xml. *T* tests assuming unequal variances were performed on mean saline and VPA log label-free quantification (LFQ) intensity values.

### Statistics

Statistics were analyzed in SPSS v.24 (IBM, Chicago, IL) and PRISM (GraphPad, La Jolla, CA) with an alpha level of 0.05. Outliers in behavioral data were removed using Grubbs’ test. Repeated measures ANOVA were run on maternal behavior counts, with drug as a between-subject factor and day as a within-subject repeated measure, and planned *t* tests with Bonferroni corrections were performed. USV counts, frequencies, and lengths were analyzed with *t* tests with Holm-Bonferroni adjustments within each sex. Fisher’s exact tests on eye opening and maternal approach counts were run. Student’s *t* tests on latency to approach and fear-potentiated startle responses for males and females were performed. For odor-potentiated startle data, a two-way drug by odor type (maternal vs neutral) ANOVA and planned *t* tests with Bonferroni correction were performed. Duration and bouts in the center of the open field were analyzed with Student’s *t* tests. Two-way drug by zone ANOVAs and planned *t* tests with Bonferroni correction were run separately for males and females for duration and bouts in the social preference (zones = social, object, empty) and social novelty (zones = novel, familiar, empty) tests. A two-way drug by decibel level ANOVA was run for baseline startle amplitude data separately for males and females, with Bonferroni-corrected *t* tests at each decibel level.

## Results

### Developmental disruption of social preference, fear expression, and anxiety-like behavior

**Fig. 1:**
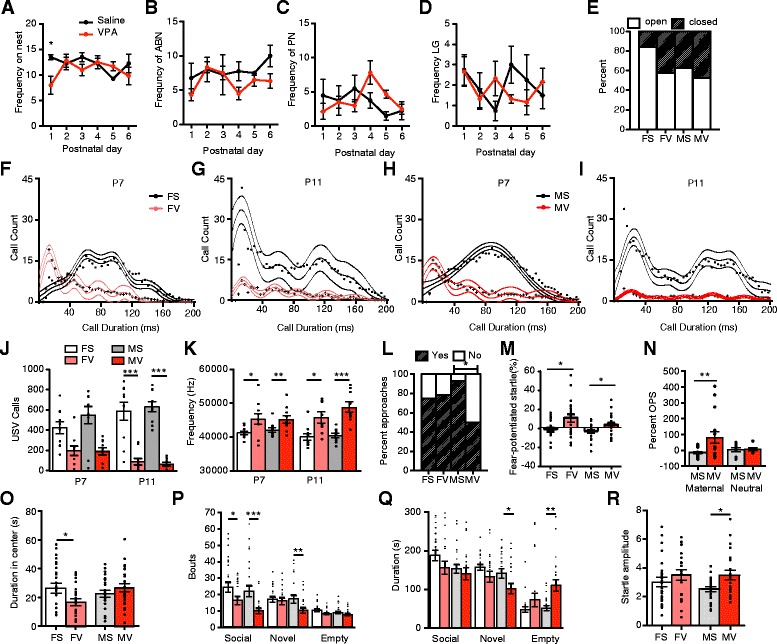
Behavioral phenotype of prenatally exposed VPA animals. Frequencies on the nest, arch-back nursing (ABN), passive nursing (PN), and licking and grooming (LG) were largely unaltered between saline- and VPA-treated dams, with the exception of P1 frequencies on the nest (*t* test with Bonferroni correction, *n* = 4–6/grp (**a**–**d**). No differences in eye openings were detected at P14 (Fisher’s exact test, females, *p* = 0.088; *n* = 19–25/grp; (**e**)). Histograms of ultrasonic vocalization lengths display qualitative differences in calls of various lengths over time and between treatment groups of each sex (*n* = 10/grp; (**f**–**i**)), with females having significantly reduced mean call lengths at P7 (not displayed, *t* test with Holm-Bonferroni correction, *p* < 0.01). VPA-treated males and females called significantly less than saline animals at P11 (**j**) and had higher frequency calls ((**k**); *t* tests with Holm-Bonferroni correction). VPA-treated males were less willing to approach maternal bedding at P9 (Fisher’s exact test; *n* = 14–16/grp; (**l**)). After fear conditioning to light at P8–10, VPA treated animals of both sexes displayed heightened fear-potentiated startle responses (*t* tests; *n* = 20–23/grp; (**m**)). Prenatal VPA treatment also enhanced startle amplitudes in the presence of maternal, but not neutral, odor (*t* tests with Bonferroni corrections; *n* = 14–17/grp, only males (**n**)). Juvenile VPA-treated females spent less time in the center of the open field (*t* test; *n* = 22–26/grp; (**o**)). Female and male VPA juveniles approached a social stimulus less than did saline males in the social preference test (post-hoc *t* tests with Bonferroni corrections; *n* = 21–26/grp; (**p**)). Males also displayed lower approaches to and less time in proximity to a novel conspecific, as well as more time in an empty arena during the social novelty component (**p**, **q**). Juvenile VPA males displayed enhanced baseline startle amplitudes (post-hoc *t* test with Bonferroni correction; *n* = 22–25/grp; (**r**)). *FS* female saline, *FV* female VPA, *MS* male saline, *MV* male VPA. Group (grp) indicates each of the four drug/sex combinations. *Asterisks* indicate significant comparisons between VPA and saline groups *p < 0.05; **p < 0.01; ***p ≤ 0.001

VPA did not lead to changes in maternal behavior in treated dams, nor did it significantly impact weight or eye opening in pups. No significant main effect of drug or drug by day interaction effect was detected for frequencies on nest, nursing, or licking and grooming (Fig. 1a–d). On P1, saline-treated dams spent more time on the nest than did VPA-treated dams (*t* test, *p* = 0.05). Weights did not differ between saline and VPA-treated animals at any age examined when combining sexes (Student’s *t* tests, *p* > 0.05; P3, 7, 14, 21, 37, 50). However, VPA-treated females weighed significantly less than their saline counterparts at P37 (*p* = 0.025). Fewer VPA females tended to open their eyes by P14 (saline 21 of 25; VPA 11 of 19; Fisher’s exact test, *p* = 0.088; Fig. 1e), but there was no difference in eye openings in males (*p* > 0.05, saline 15 of 24, VPA, 10 of 19).

Prenatal VPA impaired early communication and responses to maternal odors and enhanced fear expression in exposed pups. Mean call length of ultrasonic vocalizations was significantly shorter in prenatally exposed VPA females than in saline females at P7 (*t* test, *p* = 0.004), but not between males at P7 or either sex at P11. At P7, VPA-treated animals displayed peak call counts at 13.65 ms, compared to 76–97 ms in controls (Fig. 1f–i). VPA-treated animals called less than did saline at P11 (female *p* = 0.001, males *p* = 0.001; Fig. 1j) but not P7 (females *p* = 0.081, males *p* = 0.05), and VPA calls were of a higher frequency at both ages (P7 females *p* = 0.0028, males *p* = 0.004; P11 female *p* = 0.028, males *p* = 0.001; Fig. 1k). In the nest-seeking test, saline-treated males approached maternal bedding at a significantly higher frequency than the VPA-treated males (13 of 14 saline; 7 of 14 VPA; Fisher’s exact test, *p* = 0.033, Fig. 1l). A trend toward increased latency to approach was also observed in the VPA males (Fig. 1m, Student’s *t* test, *p* = 0.058). Females did not differ in the number that approached (saline, 12 of 16; VPA, 11 of 14). After fear conditioning to light at P8–10, VPA-treated males and females both displayed enhanced fear-potentiated startle responses to light 12 days later (*t* tests, males *p* = 0.0187; females, *p* = 0.0353). VPA males displayed enhanced startle amplitudes in the presence of the maternally conditioned odor (acetophenone) as compared to saline-treated males, but no differences were detected in responses to the neutral propanol odor (Fig. 1n; Drug X Odor ANOVA, ns; Sal vs VPA Aceto, *t* test, *p* = 0.019). Reduced distress calls, disrupted maternal nest-seeking responses, enhanced fear expression, and amplified startle responses to maternal odor indicate impaired early social behavior and enhanced fear and anxiety-like behavior in VPA-exposed males.

Prenatal VPA also enhanced anxiety-like behavior and reduced social interaction in juvenile animals. Females exposed to VPA prenatally spent less time investigating the center of the open field (Fig. 1o, Student’s *t* test, *p* = 0.031). In the social preference test, saline males and females entered the social zone more than did VPA-treated counterparts, although durations did not differ (Males; drug X zone, F_2,128_ = 4.652, *p* = 0.0112; drug, F_1,128_ = 9.682, *p* = 0.0023; Social zone *t* test, *p* < 0.001; Females; drug X zone, F_2,132_ = 4.155, *p* = 0.0178; Social zone *t* test, *p* < 0.05; Fig. 1p, q). VPA-treated males differed significantly from saline males in durations (drug X zone, F_2, 130_ = 9.872, *p* = 0.0001) and bouts (drug, F_2, 133_ = 8.78, *p* = 0.0036) in the social novelty component, spending more time in the empty arena (*p* < 0.01), less time in the arena with the novel animal (*p* < 0.05), and less bouts entering the novel arena (*p* < 0.01; Fig. 1p, q). In females, a main effect of drug was also detected for bouts in the social novelty test (F_2, 134_ = 4.156, *p* = 0.0435), but no significant effect was detected within any zone. VPA-treated males exhibited significantly larger startle responses at 110 dB noise bursts (ANOVA, drug F_2, 136_ = 7.621, *p* = 0.006 *t* test, *p* < 0.05; Fig. 1r) but no differences were detected in females. As VPA impaired social behavior and enhanced fear and anxiety-like behavior across development, and these effects were predominantly in males, genomic alterations in male neonatal and juvenile amygdala samples were examined.

### RNA sequencing

Separate Ingenuity Pathway Analyses (IPA) were run on genes with significant (*q* < 0.05) effects of time in saline (*n* = 1498) and VPA (*n* = 1682 VPA). Treatment effects did not reach statistical significance after FDR multiple comparison correction; thus, genes with uncorrected *p* < 0.05 treatment effects at P10 (*n* = 542) and P21 (*n* = 406), as well time by treatment interaction effects (*n* = 390), were run through IPA. IPA reports *p* values from a right-tailed Fisher’s exact test of the ratio of number of genes altered in a given comparison (i.e., drug, time) within the total number of genes in that pathway. Additionally, *z*-scores represent predicted changes in gene regulation of given pathways, which are based on a literature-derived Ingenuity**®** Knowledge Base [56]. Here, we focus on canonical biological pathways with activation *z*-scores and diseases and functions categories with predicted activation states (*z*-scores ≥ [2]). Additional canonical pathways with gene enrichment, without predicted activation states, can be found in the supplemental material.

**Fig. 2:**
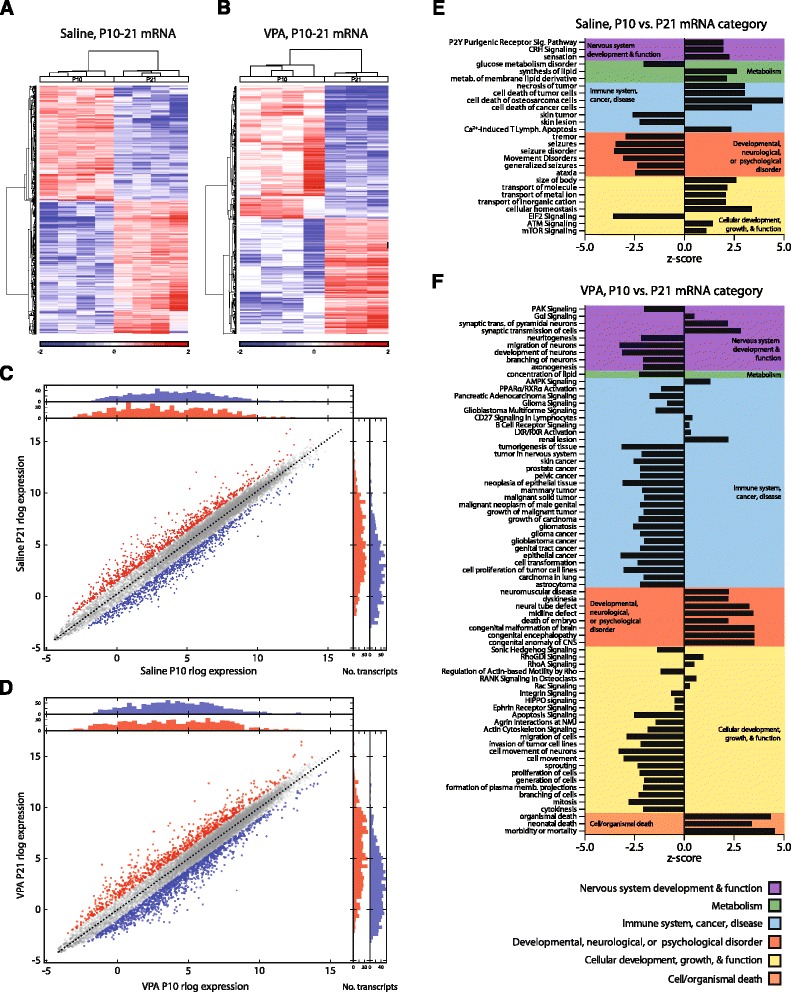
VPA disrupts cellular growth, neural development, and immune function in amygdala gene pathways from P10 to 21. Heat maps of median normalized gene expression in saline (**a**) and VPA (**b**) animals from P10 to 21 depict log2 fold changes of genes with *q* < 0.05. Differential expression scatterplots and transcript histograms of individual genes from P10 to 21 in saline (**c**) and VPA (**d**) exposed animals, where *red* and *blue points* represent genes with over 2-fold up- or down-regulation changes, respectively, in log expression across time. Ingenuity Pathway Analyses (IPA) were run on genes with significant (*q* < 0.05) effects of time in saline (*n* = 1498) and VPA (*n* = 1682 VPA) samples. Pathways with predicted activation changes from P10 to P21 exclusive to either saline (**e**) or VPA (**f**). Canonical pathways and diseases and functions categories with predicted activation or inhibition were broadly categorized into the following groups: cellular development and growth; nervous system development and function; immune system, cancer, disease; cell/organismal death; metabolism; and developmental, neurological, or psychological disorder

From P10 to P21, 72 pathways, many of which are involved in synaptic plasticity and neurotransmission, changed similarly in both saline and VPA animals (Additional file 1: Table S1A). Exclusively in saline animals, 46 pathways were altered across time, and 91 pathways were exclusively altered in the VPA group (Fig. 2, Additional file 1: Table S1B–C, 4B–C). Saline-treated amygdala samples displayed exclusive increases in pathways involved in cellular development, molecular transport, neurotransmission, and metabolism, and reductions in those involved in neurological disease. Changes from P10 to 21 unique to VPA-treated animals included alterations in pathways involved in immune responses, reductions in a number of pathways involved in cellular and neural development, and increases in pathways involved in neurological disease.

A time by treatment pathway analysis revealed a decrease in the magnitude of change in pathway activity from P10 to 21 in the VPA animals in pathways relevant to cellular development, immune function, and neurotransmission (Additional file 2: Figure S1).

**Fig. 3:**
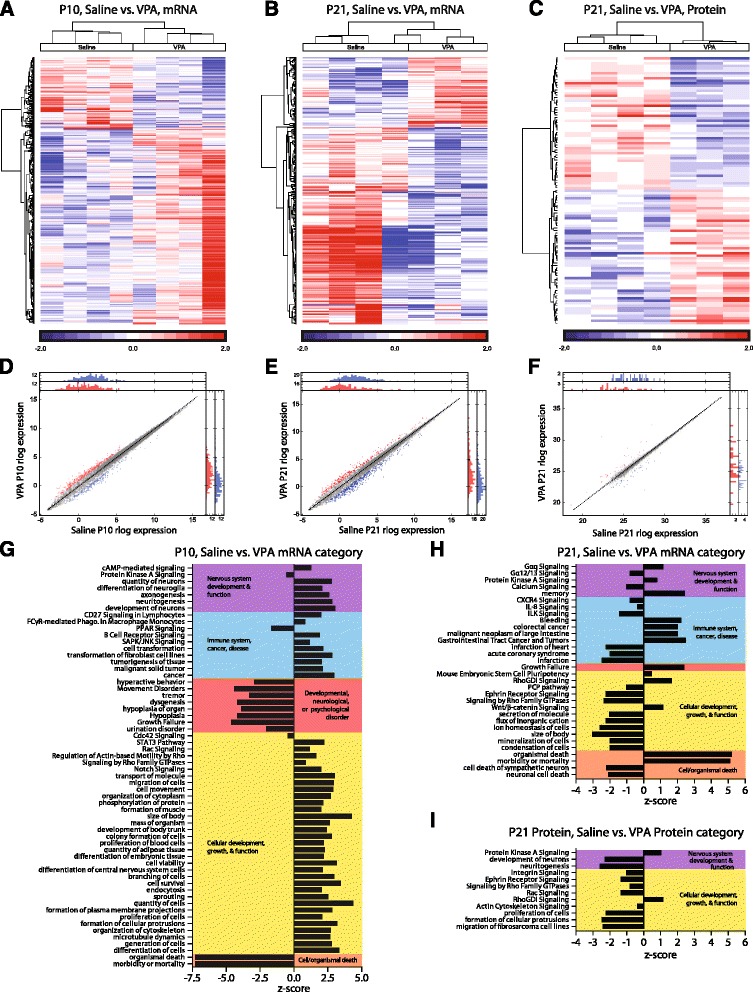
Early enhancement but later deficit in cellular growth and neural development in amygdala from prenatal VPA-exposed animals. Heat maps of differential gene expression between saline and VPA at P10 (**a**), P21 (**b**), and of differential protein expression at P21 (**c**) depict log2 fold changes of genes with *p* < 0.05. Differential expression scatterplots and transcript histograms of individual genes at P10 (**d**), P21 (**e**), and of individual proteins at P21 (**f**), where *red* and *blue points* represent molecules with over 2-fold up- or down-regulation changes, respectively, in log expression across time. Treatment effects did not reach statistical significance after FDR multiple comparison correction, thus Ingenuity Pathway Analyses were run on genes with uncorrected *p* < 0.05 treatment effects at P10 (*n* = 542) and P21 (*n* = 406). Pathways with predicted activation changes between saline and VPA transcriptomic samples at P10 (**g**) and P21 (**h**) and protein samples at P21 (**i**)

Between treatments at P10, pathways, such as those involved in cellular development and function, nervous system development and function, and immune function, were increased in the VPA-treated animals Fig. 3, Additional file 1: Table S2A, S5A). P10 VPA samples displayed predicted decreases in pathways involved in neurological and developmental diseases. By P21, there were differences in pathways involved in neurotransmission between saline and VPA amygdala samples (Additional file 1: Table S2B, S5B). Pathways involved in cellular development and function displayed decreases, and organismal death and growth failure pathways displayed increases (Fig. 3).

### Proteomics

**Fig. 4:**
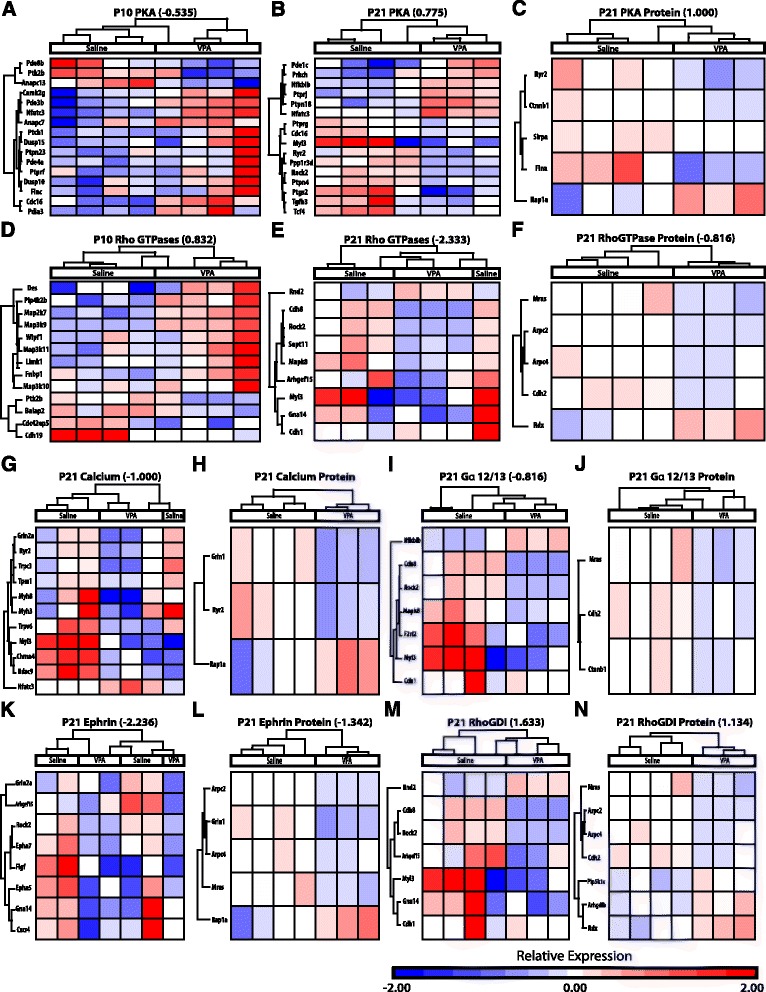
Pathways altered from prenatal VPA in both transcriptomic and proteomic samples. Individual pathway heat maps of differential gene expression between saline and VPA amygdala samples at P10 (**a**, **d**), P21 (**b**, **e**, **g**, **i**, **k**, **m**), and of differential protein expression at P21 (**c**, **f**, **h**, **j**, **l**, **n**) depict log2 fold changes of genes with *p* < 0.05. Changes in activation (*z*-scores) between groups are noted for pathways in which changes were predicted. *PKA* protein kinase A, *GDI* guanine nucleotide dissociation inhibitor

A total of 103 proteins (*p* < 0.05, uncorrected) were run in IPA analyses from proteomic results from contralateral amygdala samples from the same animals as from RNA sequencing. At P21, proteomics also revealed predicted reductions in pathways involved in the nervous system and cellular development; Additional file 1: Table S3, S6; Fig. 3). Across proteomic and transcriptomic samples from the same animals, three genes displayed alterations of *p* < 0.05 in both samples (*Ryr2*, *Slc7a14*, *Cacna2d1*). These genes also displayed the same direction of change in both assays (reductions in *Ryr2* and *Cacna2d1*, increase in *Slc7a14*). A number of pathways displayed predicted alterations across both transcriptomic and proteomic analyses at P21 (Fig. 4). Notably, PKA signaling and signaling by Rho Family GTPases are the only pathways altered between saline and VPA animals at all time points (P10, 21) and in both proteomic and transcriptomic samples.

## Discussion

Prenatal exposure to VPA disrupted later social behavior, fear expression, anxiety-like behavior, and amygdala gene expression in infant and juvenile animals, with many alterations corresponding to ASD-like symptomatology (Table 2). Maternal behavior was largely unaltered in dams given VPA, suggesting changes in offspring are not due to indirect maternal effects. A reduction in time spent in social proximity and enhancement in isolation were detected in the VPA-treated males. This reduction in sociality is in accordance with a number of previous reports of the VPA model [8, 23, 57–61]. Prenatal VPA also lead to enhancements in basal anxiety with males displaying greater startle amplitudes and females investigating the center of the open field less than saline counterparts. Enhanced baseline acoustic startle responses are correlated with anxiety disorders like PTSD [62], as well as with ASD in adolescents [63].

**Table 2:** Overlap between VPA model and ASD

ASD	Prenatal VPA model
Core Pathology [9]	
Deficits in social interaction	Reduced social investigation, impaired attraction to maternal bedding, maternal odor-induced potentiation of startle
Impaired social communication	Reduced USVs and altered call structure
Repetitive/stereotypical behaviors	Increased stereotypic-like beam breaks [8, 57]
Additional Symptoms	
Comorbid anxiety disorders [36, 37]	Reduced open field exploration in females Enhanced baseline startle amplitude in males
Early amygdala overgrowth [32]	Enhanced P10 nervous system and cellular development and function pathways
Reduced amygdala volume [11, 12], neuron number [15], and activity [19, 20] in adolescents and adults	Reduced P21 nervous system and cellular development and function, and enhanced cellular death and psychological disorder pathways
Gastrointestinal problems [97]	Enhanced P21 gastrointestinal tract and colorectal cancer genes
Immune alterations [96]	Altered P10 and P21 immune function and cancer pathways
Male predominance [64]	Behavioral alterations primarily in males

Notably, behavioral effects were found primarily in male rats, paralleling the male predominance of ASD and neurodevelopmental disorders resulting from prenatal VPA [3, 64, 65]. Sex differences have been detected in some [57, 60, 66, 67] but not all [59] previous studies of the VPA animal model in which sexes were compared. Impairments in social behavior that are enhanced or exclusive to male VPA-exposed offspring have been reported in rats and mice [32, 57, 60]. Reduced pain sensitivity, enhanced anxiety-like behavior in an elevated plus maze, and increased cortical and hippocampal post-synaptic marker proteins have also been detected exclusively in males [57, 60]. Raza et al. [67] found that prenatal VPA exposure impaired motor behavior and decreased closed-arm time exclusively in female rats. Stefánik et al. [68] reported increased social interaction in females prenatally exposed to VPA. However, Roullet et al. [59] reported male and female mice exposed prenatally to VPA were similarly impaired in sociability scores and nest-seeking responses. An increase in social investigation was reported by Cohen et al. [69]; however, a lower 350 mg/kg dose was used and sexes were combined in the analyses. In other studies, sexes were combined in analyses with no reports of sex differences [23, 70] or only male behavioral deficits were examined [8, 58, 61, 71, 72]. Due to enhanced behavioral deficits observed in males in our sample and previous reports [32, 57, 60], genomic analyses were run in male amygdala. However, future investigation into potential protective pathways in females that accounts for reduced deficits warrants future investigation.

The amygdala undergoes significant functional, morphological, and physiological maturation during the first 2 weeks of development, and insults to this region can dramatically alter later socioemotional behavior. By postnatal day 10, the ability to learn fear associations emerges, which corresponds to changes in amygdala including differential responsivity to shock [42], enhanced synaptic plasticity [44], an emergence of dendritic spines [43], and a switch in the postsynaptic GABA response from excitation to inhibition [39]. Significantly, learned fear in young rats (P17) is no longer expressed after 10 days, but early maternal separations induce adult fear retention [73]. Prenatal VPA exposure may similarly have led to early maturation of fear responses, which is supported by the observed enhancements in fear expression after infant fear conditioning in our data. A hyperactive, precocious amygdala may lead to an early termination of the attachment-learning period and the beginning of fear learning [74].

Early in development, neonates experience a sensitive period for attachment learning and impaired fear learning, thought in part to be mediated via maternal suppression of pup corticosterone levels [75]. VPA-treated males displayed reduced approaches to maternal bedding and increased startle amplitudes in the presence of the maternal odor, and both sexes displayed reduction in distress ultrasonic vocalizations upon separation from the mother. Reduced vocalizations and altered call structures (reduced length, increased frequency) are suggestive of impaired social communication and imply a functional difference in these calls, which may further exacerbate social impairments through development. The number and length of calls increase after an isolated pup is briefly reunited with the mother, known as maternal potentiation [76, 77]. Furthermore, anxiolytics and antidepressants reduce call length in neonates during isolation [78], suggesting the decreased duration and rate of calls in VPA pups may indicate reduced distress from maternal separation. Increased infant call frequencies were also observed in VPA pups, a pattern previously associated with reduced fitness and increased risk for ASD [79, 80]. Olfactory learning is particularly important for attachment during this time period, and the quality of the infant-mother bond and early olfactory learning is a salient predictor of later social relationships [81]. Learned maternal odors serve as safety signals later in life, reducing depressive-like behavior, attenuating fear conditioning, and enhancing social behavior in rats exposed to early stress [82, 83]. Rather than attenuating startle, the presence of the maternal odor (acetophenone) enhanced baseline startle responses, while not impacting responses in control animals. It should be noted that female startle amplitudes in response to maternal odor were not measured and warrant future investigation. The observed alterations in prenatal VPA-treated pup behavior may reflect abnormal amygdala functioning and maternal-infant bonding.

**Fig. 5:**
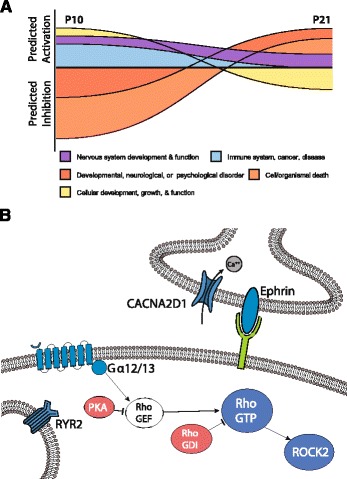
Alterations in amygdala genomic profile resulting from prenatal VPA exposure. Pathway changes predicted in animals exposed to VPA prenatally differed across time, with enhancements in developmental and growth pathways predicted early and reductions predicted later in development (**a**). Predicted reductions (*blue*) or enhancements (*red*) common to P21 proteomic and transcriptomic analyses were observed in pathways involved in neurotransmission and synaptic plasticity (**b**). G-protein coupled receptor activation of Gα12/13 proteins leads to activation of Rho GTPases by dissociating from the inhibitory RhoGDI, and one pathway is through the release of the inhibitory PKA from RhoGEF [110]. Reductions in ROCK2, a downstream effector that inhibits dendritic remodeling, may lead to structural changes in dendritic spines in VPA animals. VPA-exposed animals displayed impaired ephrin receptor and calcium signaling pathways, with reduced *Ryr2* and *Cacna2d1* expression in both transcriptomic and proteomic analyses. *CACNA2D1* voltage-gated calcium channel auxiliary subunit, *RYR2* ryanodine receptor 2, *PKA* protein kinase A, *GEF* guanine nucleotide exchange factor, *GDI* guanine nucleotide dissociation inhibitor, *ROCK2* Rho kinase 2

VPA, a nonselective histone deacetylase inhibitor, may transiently increase histone acetylation in the developing embryonic brain when exposed in utero [66], leading to widespread changes in the amygdala transcriptome. This is the first analysis of transcriptomic and proteomic changes across development in the amygdala in both normative and VPA-exposed conditions. Many pathways involved in development, nervous system function, and the immune system displayed predicted activations in VPA amygdala samples at P10, whereas at P21 pathways involved in cell death and developmental disorders predominated (Fig. 5a). Across time, VPA appears to be stunting the normal developmental alterations in amygdala gene expression from P10 to P21. A number of pathways involved in synaptic plasticity, neurotransmission, cellular growth, immune function, and metabolism are reduced in VPA-exposed animals across time relative to saline-treated animals. Conversely, pathways involved in cell death and neurological and developmental disorders are increased across time exclusively in the VPA group. Previous microarray analyses of adult amygdala gene expression from prenatally exposed VPA animals pointed to alterations in similar pathways, such as neuronal projection, cell-cell signaling, synaptic transmission, vesicle, and calcium signaling pathways [69]. Moreover, a microarray study from 35-day-old rats exposed to VPA on E12 reported that the amygdala displayed alterations in many similar IPA gene pathways, including cell death, cell signaling, development, proliferation, movement, inflammatory disease, molecular transport, neurological disease, developmental, psychological disorder, and tissue development [84]. It should be noted that although changes in gene expression across development within each group survived multiple comparisons, differences between the groups did not, and thus these results should be considered exploratory analyses to identify targets for future investigation. Furthermore, the BLA was specifically targeted at P21, but P10 samples may include surrounding amygdala regions.

These data are remarkably in line with transcriptomic analysis of the temporal and frontal cortex showing alterations in genes involved in immune function and synaptic plasticity between individuals with and without ASD [85]. A gene co-expression module involved in synaptic function, vesicular transport, and neuronal projection was downregulated, whereas a module associated with astrocyte markers, activated microglia, immune and inflammatory responses was upregulated in ASD adult samples. The authors also observed an attenuation of gene expression differences between frontal and temporal cortices. Previous microarray-based analyses have revealed similar alterations in developmental or immune pathways in ASD [86–88]. Chow et al. [87] examined dorsolateral prefrontal cortex gene expression in both young and adult brains of individuals with ASD and reported alterations in pathways involved in neurogenesis, neurodevelopment, cell cycle, DNA damage response, apoptosis, cytoskeleton remodeling, and the immune response. ASD susceptibility genes involved neurodevelopmental pathways such as neuritogenesis, axogenesis, quantity of axons, and formation of neural tube display prenatal enrichment in the dorsolateral prefrontal cortex [88]. Transcriptomic analyses have also found enrichment of ASD genes in cortical projection neurons during embryonic development [89, 90], suggesting future analyses of the cortex are warranted at P10, the rat equivalent of the third trimester in humans [91]. A previous microarray analyses in rats revealed differing effects of VPA on OFC, as compared to anterior amygdala and cerebellar vermis gene expression, which were more similar [69]. However, in a study of primate neuro-development using laser-capture microdissection and microarray profiling from early gestation (E50) through the first 48 months, the amygdala had both an earlier onset of genes related to synaptogenesis and myelination, and a quicker downregulation of those same processes compared to basal ganglia, hippocampus, and neocortex [92]. Genes related to ASD were also enriched in early developing regions like the amygdala, as opposed to genes related to schizophrenia which saw increased expression post birth. Together, these data would suggest that gene disruption in response to fetal VPA exposure will be regulated not only by the process of normative cellular development but also by the time at which individual components of circuits involved in affective regulation come “on-line”.

At P10, pathways involved in the development, morphology, proliferation, and differentiation of cells displayed predicted increases in the amygdala of males exposed to VPA in utero. Increases in pathways involved in neuronal signaling and immune function, and decreases in those involved in developmental disorders were predicted in VPA animals. An early enhancement in cellular differentiation and proliferation is in line with an early overgrowth of the amygdala in ASD [32–34] and may underlie the observed enhancement in fear learning in infants exposed to VPA. Indeed, VPA promotes neurogenesis and cortical neuronal growth in primary cultures [93].

A number of pathways were similarly altered across P21 transcriptome and proteome samples (Figs. 4 and 5b). Although more immediate effects of VPA in early development may appear beneficial, abnormalities appeared later in the development. In juvenile P21 males exposed to VPA prenatally, a number of amygdala gene pathways involved in neurotransmission, cellular development, nervous system development, cell death, and immune function displayed alterations. Both proteomic and transcriptomic analyses of contralateral amygdala from the same animals at P21 revealed reductions in pathways involved in cellular development and proliferation, which is in contrast to the enhancement in these pathways found at P10. Both transcriptomic and proteomic analyses revealed reduced activity of the ephrin receptor signaling pathway, which regulates axonal guidance and cell migration early in development. Over time from P10 to 21, the ephrin pathway was reduced exclusively in the VPA animals. Knockouts in the ephrin pathway cause ASD-like symptoms [94]. Interestingly, although early premature overgrowth of the amygdala is described in ASD [32–34], reductions in amygdala volume [11, 12], neuron number [15], and functional activity [16–19] are observed later in life.

A predicted reduction in activity of immune function pathways was detected in P21 proteomic and transcriptomic samples, as well as in transcriptomic samples from P10 to 21, in VPA animals, which is in line with previously reported immune alterations in VPA rats [57]. Interleukin function has been correlated to social behavior and ASD [95]. Enhanced autoimmunity, allergies, asthma, and reduced immune function have been reported in individuals with ASD [96]. Enhancements in gastrointestinal tract and colorectal cancer were predicted at P21, which is in line with commonly reported gastrointestinal problems in ASD [97]. Reductions in natural killer cell function [98], increases in chemokines, and enhancements in pro-inflammatory cytokines have been associated with ASD [99, 100]. As cytokines serve dual functions in both the immune system and in fetal brain development [101], immune regulators may underlie impairments in both.

The calcium signaling pathway was significantly altered in proteomic and transcriptomic analyses, with reduced activity in VPA animals predicted by mRNA. Between P10 and 21, both saline and VPA amygdala displayed predicted increases in the calcium signaling pathway, however, to a greater extent in the saline group. Ryanodine receptor 2 (*Ryr2*) and the Calcium Voltage-Gated Channel Auxiliary Subunit α2δ 1 (*Cacna2d1*) were two of the three genes altered in both mRNA and protein analyses and both displayed reduced expression across analyses. Notably, *RYR2*, which mediates sarcoplasmic calcium release, is a potential ASD risk gene in humans [102, 103]. *RYR2* mRNA is differentially expressed between the frontal and temporal cortices in postmortem tissue from control individuals, but not those with ASD [85]. *Cacna2d1* promotes calcium-induced neurotransmitter exocytosis through regulating voltage-gated calcium channel trafficking and increased efficiency of exocytosis [104]. Significantly, functional mutations in several genes encoding voltage-gated calcium channels have also been linked to ASD (see [105]). Alteration in calcium signaling, and thus neuronal communication, may contribute to aberrant amygdala functioning and thus of behavior in VPA rats and individuals with ASD.

Pathways altered across proteomic and transcriptomic analyses at both developmental time points were protein kinase A (PKA) signaling and signaling by Rho family GTPases. A reduction in PKA signaling at P10 and an increase at P21 in VPA were predicted relative to saline amygdala samples. Overtime, both groups displayed predicted increases in the PKA pathway. PKA signaling regulates emotionality and social anxiety, and dysfunction in this pathway is implicated in ASD [106, 107]. Alterations in PKA may underlie changes in synaptic plasticity and activity seen in the amygdala of VPA-exposed animals. We have previously shown that PKA signaling in the amygdala is critical for dopamine D1 receptor facilitation of long-term potentiation [108], enhances membrane potential oscillations in BLA principal neurons [48], and bidirectionally controls synaptic strength [47]. Membrane potential oscillations enhance spike-timing precision and coordinated firing of BLA principal neurons [48], thus VPA-induced changes in PKA activity may significantly alter functional activity of the amygdala.

An increase in signaling by Rho family GTPases at P10, but a reduction at P21, was predicted in VPA amygdala. Over time, this pathway displayed a predicted increased in saline animals but a decrease in VPA exposed males. RhoGTPases are essential regulators of neuronal motility and morphology, particularly in the development and maturation of dendritic spines, and alterations in these systems have been linked to ASD [109]. GTP bound Rho activates Rho-kinase 2 (ROCK2), which ultimately inhibits neurite outgrowth and promotes cell contraction (for review see [110, 111]). Down-regulation of Rho pathways at P21 may contribute for amygdala hyperplasticity in the VPA model [23, 24]. However, knockout of *Rock2* expression reduces dendritic spine density and synaptic transmission [112]. Thus, functional outcomes of altered Rho signaling in juvenile animals warrant future investigation. Interestingly, the Rho-Guanine nucleotide dissociation inhibitor (GDI) signaling pathway, which inhibits Rho family GTPases, displayed a corresponding predicted enhancement in activity at P21 in both transcriptomic and proteomic analyses. Additionally, the RhoGDI pathway displayed an exclusive increase in activity in VPA animals.

It should be noted that previous work has found very modest overlap between transcriptomic and proteomic samples, which may be due to posttranscriptional processing such as alternative splicing, modifications, translational efficacy, and degradation [113]. In a comprehensive genomic analysis of mouse liver tissue, an average correlation of only 0.27 was obtained between levels of transcripts and proteins [114]. Similarly, an analysis of human brain tissue yielded only 0.25 mRNA-protein correlation [115]. When controlling for neural cell type, a maximum of 0.47 correlation was reached [116]. We are also limited in mRNA-protein comparisons in that transcriptomic analyses mapped reads to ~17,000 genes, whereas significantly less proteins were assessed (~3500). Future targeted quantification of differentially expressed gene proteins, especially ones highlighted in Fig. 5b that overlapped in transcriptomic and proteomic assays, should be performed, as well as cell-type specific or single cell genomic approaches.

## Conclusions

These exploratory genomic analyses provide potential targets of interest in the amygdala in the study of molecular underpinnings of ASD. Furthermore, behavioral and genetic alterations observed support the use of prenatal VPA exposure as an effective tool for the study of pathways underlying social dysfunction relevant to ASD. Future studies should investigate gene expression and physiology of individual BLA neuronal populations throughout development in animals prenatally exposed to VPA. An understanding of the genetic alterations occurring in BLA neurons could identify potential drug targets and critical windows for treatment interventions for anxiety in children with ASD.

## Additional files

Additional file 1: Table S1-6. **Table S1.** Canonical RNA sequencing pathways differing from P10 to 21 in (A) both VPA and saline amygdala, (B) exclusively in saline amygdala, or (C) exclusively in VPA amygdala. **Table S2.** Canonical RNA sequencing pathways differing between saline and VPA amygdala at (A) P10 and (B) P21. **Table S3.** Canonical Proteomic Pathways differing between saline and VPA amygdala at P21. **Table S4.** Diseases and Functions RNA Sequencing Categories differing from P10 to 21 in (A) both VPA and saline amygdala, (B) exclusively in saline amygdala, or (C) exclusively in VPA amygdala both VPA and saline amygdala. **Table S5.** Diseases and functions RNA sequencing categories differing between saline and VPA amygdala at (A) P10 and (B) P21. **Table S6.** Diseases and functions proteomic categories differing between saline and VPA amygdala at P21. (DOCX 86 kb)

Additional file 2: Figure S1. RNA sequencing pathways differentially altered across development between VPA and saline amygdala. Pathways with significant time (P10–21) by treatment (VPA/saline) effects are displayed. Treatment effects did not reach statistical significance after FDR multiple comparison correction, thus Ingenuity Pathway Analyses were run on genes with uncorrected *p* < 0.05 treatment effects at P10 (*n* = 542) and P21 (*n* = 406). Canonical pathways and diseases and functions categories with predicted activation or inhibition differences were broadly categorized into the following groups: cellular development and growth; nervous system development and function; immune system, cancer, disease; cell/organismal death; metabolism; and developmental, neurological, or psychological disorder. (PDF 59 kb)

## Abbreviations

ASD: Autism spectrum disorder
BLA: Basolateral amygdala
IPA: Ingenuity Pathway Analysis
LC-MS/MS: Liquid chromatography–tandem mass spectrometry
PKA: Protein kinase A
Rho-GDI: Rho-Guanine nucleotide dissociation inhibitor
ROCK2: Rho-kinase 2
RYR2: Ryanodine receptor 2
USV: ultrasonic vocalization
VPA: valproic acid
