# Supplementary material for: Developmental disruption of amygdala transcriptome and socioemotional behavior in rats exposed to valproic acid prenatally
Source: Mol Autism. 2017 Aug 1;8:42. doi: 10.1186/s13229-017-0160-x (PMC5539636; doi:10.1186/s13229-017-0160-x)
Supplement: Supplementary file 1 — Table S1. Canonical RNA sequencing pathways differing from P10 to 21 in (A) both VPA and saline amygdala, (B) exclusively in saline amygdala, or (C) exclusively in VPA amygdala. Table S2. Canonical RNA sequencing pathways differing between saline and VPA amygdala at (A) P10 and (B) P21. Table S3. Canonical Proteomic Pathways differing between saline and VPA amygdala at P21. Table S4. Diseases and Functions RNA Sequencing Categories differing from P10 to 21 in (A) both VPA and saline amygdala, (B) exclusively in saline amygdala, or (C) exclusively in VPA amygdala both VPA and saline amygdala. Table S5. Diseases and functions RNA sequencing categories differing between saline and VPA amygdala at (A) P10 and (B) P21. Table S6. Diseases and functions proteomic categories differing between saline and VPA amygdala at P21. (DOCX 86 kb) [file 13229_2017_160_MOESM1_ESM.docx]

**Additional file 1**

Table S1. Canonical RNA Sequencing Pathways differing from P10-21 in (A) both VPA and Saline amygdala, (B) exclusively in Saline amygdala or (C) exclusively in VPA amygdala. Ratio represents the number of genes altered between treatments within the total number of genes in that pathway, and the p-value is from a right-tailed Fisher exact test of this ratio. The z-score represents the predicted change in gene regulation of that pathway.

A.

| *Group* | *Comparison* | *Ingenuity Canonical Pathways* | *-log(p-value)* | *Ratio* | *z-score* | *Molecules* |
| --- | --- | --- | --- | --- | --- | --- |
| VPA | P10-21 | 14-3-3-mediated Signaling | 2.57 | 0.144 |  | RAF1,TUBB3,MAPK1,TUBB2A,MAPK8,TUBB,PRKCZ,TUBB2B,SRPK2,PLCB4,TUBA1A,PLCE1,TUBB6,KL,MAPT,PLCB1,TUBA1C,TUBB4A,MAP2K1 |
| Saline | P10-21 |  | 4.03 | 0.159 |  | TUBB3,MAPK1,PDIA3,TUBB2A,TUBA4A,BAX,TUBB,PRKCZ,TUBB2B,PRKCG,SRPK2,PLCB4,TUBA1A,TUBB6,MAPT,MAPK10,PLCB1,TUBA1C,TUBB4A,PDCD6IP,MAP2K1 |
| VPA | P10-21 | Aldosterone Signaling in Epithelial Cells | 1.57 | 0.114 | 0.277 | RAF1,CRYAB,MAPK1,DNAJB4,HSPH1,DNAJC27,PIP4K2B,ITPR1,PRKCZ,PLCB4,PLCE1,KL,ASIC1,PLCB1,NR3C2,MAP2K1,PIP4K2C,DNAJB5,HSPB6,HSPA4L |
| Saline | P10-21 |  | 2.37 | 0.119 | 2.714 | CRYAB,MAPK1,DNAJC9,PDIA3,DNAJB4,HSPH1,DNAJC27,PDPK1,ITPR1,PRKCZ,PRKCG,PLCB4,PIP5K1A,DUSP1,PLCB1,MAP2K1,DNAJC16,PIP4K2C,DNAJB5,HSPB6,HSPA4L |
| VPA | P10-21 | Amyotrophic Lateral Sclerosis Signaling | 4.42 | 0.183 |  | PRPH,CAPN5,GRIN2A,CAPN6,CASP3,GRIN2D,GRIA2,APAF1,SOD1,GRIN3A,GRINA,BCL2L1,GRIK5,IGF1,KL,GLUL,SLC1A2,CAPN2,CASP7,PPP3CA,GRIA3,GRIK1 |
| Saline | P10-21 |  | 2.39 | 0.133 |  | GRIN2A,CAPN6,CASP3,GRIN2D,APAF1,BAX,GRINA,BCL2L1,IGF1,GRIK4,GLUL,SLC1A2,CASP7,PPP3CA,GRIA3,GRIK1 |
| VPA | P10-21 | Atherosclerosis Signaling | 2.01 | 0.133 |  | PLA2G16,ALOX15,PDGFA,COL2A1,F3,ALOXE3,SELPLG,IL33,ALB,LYZ,ALOX15B,IL1RN,ALOX5,CLU,RBP4,APOD,PAFAH1B3 |
| Saline | P10-21 |  | 1.78 | 0.117 |  | PLA2G16,COL2A1,CXCL12,F3,ALOXE3,SELPLG,PLA2G6,ALB,LYZ,IL1RN,COL11A2,ALOX5,RBP4,APOD,PAFAH1B3 |
| VPA | P10-21 | Axonal Guidance Signaling | 6.84 | 0.141 |  | DPYSL2,RAF1,RAC2,MAPK1,Wasl,GNB5,TUBB,LIMK1,ROCK2,PTK2,SEMA6D,PLCE1,ECE2,PPP3R1,ABLIM3,BAIAP2,WNT4,PLCB1,ABLIM2,TUBA1C,EFNB3,TUBB3,NGEF,PAPPA,STK36,SEMA5A,TUBB2A,PTCH1,L1CAM,HHIP,DPYSL5,ADAMTS9,HERC2,NRP1,ADAMTS7,PLXNA3,SLIT1,PDGFA,ARHGEF7,SEMA6C,PRKCZ,TUBB2B,IGF1,KL,SRGAP1,MKNK1,TUBB4A,SEMA3B,MAP2K1,PPP3CA,ITGB1,C9orf3,ROCK1,GNAI3,PLCB4,TUBA1A,TUBB6,GLIS2,PAK3,EPHA5,SEMA4G,EPHB3,SEMA3C,GLI1 |
| Saline | P10-21 |  | 2.64 | 0.0991 |  | SLIT3,ADAMTS7,PLXNA3,ECEL1,MAPK1,PDIA3,Wasl,CXCL12,GNB5,GNG13,TUBB,SEMA6C,PRKCZ,TUBB2B,ROCK2,PTK2,IGF1,PPP3R1,BAIAP2,ABLIM3,PRKAR1B,UNC5D,PLCB1,ABLIM2,TUBA1C,TUBB4A,SEMA3B,EFNB3,MAP2K1,PPP3CA,TUBB3,CRKL,C9orf3,TUBB2A,TUBA4A,L1CAM,HHIP,DPYSL5,PRKCG,GNAI3,PLCB4,SEMA4D,TUBA1A,TUBB6,SEMA3C |
| VPA | P10-21 | Breast Cancer Regulation by Stathmin1 | 8.03 | 0.191 |  | RAF1,CAMK4,MAPK1,ARHGEF7,GNB5,PPP1CB,TUBB,PPP1R14B,PRKCZ,LIMK1,TUBB2B,STMN1,ROCK2,CAMK2A,ADCY5,PPP1R7,KL,PLCB1,TUBB4A,TUBA1C,PPP2R2C,ARHGEF3,ADCY8,MAP2K1,PPP1R14C,TUBB3,TUBB2A,PPP1R11,ITPR1,CDK1,ROCK1,GNAI3,PLCB4,TUBA1A,TUBB6,PPP2R3A,ADCY10,ARHGEF9,CDK2,CAMK2G |
| Saline | P10-21 |  | 7.84 | 0.177 |  | CAMK4,MAPK1,GNB5,GNG13,TUBB,PPP1R14B,PRKCZ,TUBB2B,ROCK2,STMN1,CAMK2A,ADCY5,PRKAR1B,PLCB1,TUBB4A,PPP2R2C,TUBA1C,ARHGEF3,ADCY8,MAP2K1,PPP1R14C,TUBB3,TUBB2A,TUBA4A,PPP1R14A,PPP1R11,ITPR1,CDK1,PRKCG,GNAI3,ADCY9,PLCB4,TUBA1A,TUBB6,PPP2R3A,ARHGEF9,CDK2 |
| VPA | P10-21 | Calcium Signaling | 3.84 | 0.151 | 0.426 | GRIN2A,CAMK4,MAPK1,GRIN2D,CHRNB1,Tpm1,TRPC3,GRINA,CAMK2A,HDAC11,PPP3R1,CHRNA3,PPP3CA,GRIK1,AKAP5,CHRNA4,TNNC2,SLC8A3,GRIA2,ITPR1,TRPC7,GRIN3A,MICU1,CAMKK1,MYH3,SLC8A1,CAMK2G,GRIA3 |
| Saline | P10-21 |  | 1.81 | 0.108 | 2.828 | AKAP5,GRIN2A,CAMK4,MAPK1,TNNC2,GRIN2D,RYR2,ITPR1,GRINA,TRPC7,MICU1,CAMK2A,PPP3R1,MEF2D,CAMKK1,PRKAR1B,PPP3CA,CAMKK2,GRIK1,GRIA3 |
| VPA | P10-21 | cAMP-mediated signaling | 6.65 | 0.173 | 2.744 | AKAP12,ADRA2B,GPR17,RAF1,CAMK4,AKAP8,HTR4,MAPK1,PDE1A,HRH3,CAMK2A,DRD1,ADCY5,PPP3R1,SMPDL3B,RGS14,PKIA,ADCY8,MAP2K1,HCAR2,PPP3CA,HTR6,AKAP5,RGS2,NPY1R,MC4R,PDE1C,GNAI3,GABBR2,LPAR1,CREM,PDE1B,S1PR1,PDE8B,HTR1F,ADRA2C,ADCY10,ADORA2A,CAMK2G |
| Saline | P10-21 |  | 3.97 | 0.133 | 2.041 | AKAP12,CAMK4,AKAP8,MAPK1,CHRM4,PDE1A,HRH3,AKAP11,CAMK2A,ADCY5,PPP3R1,HTR7,PRKAR1B,PKIA,ADCY8,MAP2K1,PPP3CA,ADRB2,AKAP5,PDE2A,RGS4,CNGA1,GNAI3,ADCY9,TULP2,LPAR1,DUSP1,CREM,PDE1B,S1PR1 |
| VPA | P10-21 | Cardiac Hypertrophy Signaling | 1.35 | 0.102 | 0.408 | ADRA2B,MAP3K9,RAF1,CAMK4,MAPK1,MAPK8,GNB5,ROCK2,ROCK1,MAP3K10,GNAI3,PLCB4,PLCE1,RHOG,IGF1,RND3,KL,ADCY5,PPP3R1,PLCB1,ADRA2C,ADCY8,ADCY10,MAP2K1,PPP3CA |
| Saline | P10-21 |  | 1.4 | 0.0943 | 2.837 | CAMK4,MAPK1,PDIA3,GNB5,GNG13,ATF6,ROCK2,ADCY9,GNAI3,PLCB4,RHOG,IGF1,RND3,ADCY5,PPP3R1,MEF2D,MAPK10,PRKAR1B,PLCB1,ADCY8,MAP2K1,PPP3CA,ADRB2 |
| VPA | P10-21 | Cardiac β-adrenergic Signaling | 3.28 | 0.154 | 1.5 | AKAP12,AKAP5,PPP1R14C,AKAP8,GNB5,SLC8A3,PPP1CB,PPP1R11,PDE1A,PPP1R14B,PDE1C,PPP1R7,ADCY5,PPP2R3A,PDE1B,PPP2R2C,PDE8B,SMPDL3B,PKIA,ADCY8,SLC8A1,ADCY10 |
| Saline | P10-21 |  | 3.97 | 0.154 | 1.807 | AKAP12,AKAP5,PPP1R14C,PDE2A,AKAP8,RYR2,GNB5,GNG13,PPP1R14A,PPP1R11,PDE1A,PPP1R14B,AKAP11,ADCY9,TULP2,ADCY5,PPP2R3A,PDE1B,PRKAR1B,PPP2R2C,PKIA,ADCY8 |
| VPA | P10-21 | CDK5 Signaling | 5.52 | 0.214 | 0.655 | ITGB1,RAF1,LAMA5,PPP1R14C,MAPK1,PPP1R1B,EGR1,MAPK6,MAPK8,PPP1CB,PPP1R11,PPP1R14B,DRD1,PPP1R7,ADCY5,PPP2R3A,MAPT,LAMA1,PPP2R2C,ADCY8,ADCY10,MAP2K1 |
| Saline | P10-21 |  | 4.07 | 0.175 | 0.471 | PPP1R14C,MAPK1,MAPK4,EGR1,MAPK6,PPP1R14A,PPP1R11,PPP1R14B,ADCY9,PPP2R3A,ADCY5,MAPT,PRKAR1B,MAPK10,LAMA1,PPP2R2C,ADCY8,MAP2K1 |
| VPA | P10-21 | Cell Cycle Control of Chromosomal Replication | 5.09 | 0.316 |  | MCM5,MCM3,TOP2B,LIG1,MCM6,MCM2,POLE,CDC6,ORC5,TOP2A,POLA2,CDK2 |
| Saline | P10-21 |  | 5.6 | 0.316 |  | MCM5,MCM3,TOP2B,LIG1,MCM6,MCM2,POLE,ORC5,ORC6,CDK4,TOP2A,CDK2 |
| VPA | P10-21 | Cell Cycle: G2/M DNA Damage Checkpoint Regulation | 3.25 | 0.224 | 1.897 | TOP2B,CDC25C,TOP2A,CCNB2,PKMYT1,RPRM,AURKA,CDK1,PRKCZ,SKP2,CCNB1 |
| Saline | P10-21 |  | 3.67 | 0.224 | 2.53 | TOP2B,CDC25C,WEE1,TOP2A,CCNB2,PLK1,CDK1,PRKCZ,SKP2,CHEK1,CCNB1 |
| VPA | P10-21 | Cellular Effects of Sildenafil (Viagra) | 1.86 | 0.126 |  | CACNG1,CAMK4,PPP1CB,ITPR1,PRKG2,PDE1A,PDE1C,CACNG2,PLCB4,PLCE1,GPR37,ADCY5,PDE1B,MYH3,PLCB1,ADCY10,ADCY8,SLC4A10 |
| Saline | P10-21 |  | 1.4 | 0.105 |  | PDE2A,CAMK4,PDIA3,SLC4A11,ITPR1,PDE1A,ADCY9,PLCB4,GPR37,ADCY5,PDE1B,PRKAR1B,PLCB1,ADCY8,SLC4A10 |
| VPA | P10-21 | Chemokine Signaling | 3.63 | 0.2 | 0.258 | RAF1,CAMK4,PTK2B,MAPK1,MAPK8,PPP1CB,LIMK1,ROCK2,PTK2,GNAI3,PLCB4,CAMK2A,PLCB1,MAP2K1,CAMK2G |
| Saline | P10-21 |  | 2.11 | 0.147 | 2.111 | ROCK2,PTK2,GNAI3,PLCB4,CAMK4,CAMK2A,MAPK1,PTK2B,CXCL12,PLCB1,MAP2K1 |
| VPA | P10-21 | Cholecystokinin/Gastrin-mediated Signaling | 2.86 | 0.16 |  | RAF1,PTK2B,MAPK1,MAPK8,ITPR1,PRKCZ,IL33,ROCK2,PTK2,ROCK1,PLCB4,RHOG,RND3,IL1RN,CREM,PLCB1,MAP2K1 |
| Saline | P10-21 |  | 3.42 | 0.16 |  | PTK2B,MAPK1,ITPR1,PRKCZ,PRKCG,ROCK2,PTK2,PLCB4,RHOG,RND3,IL1RN,MEF2D,CREM,MAPK10,PLCB1,PTGS2,MAP2K1 |
| VPA | P10-21 | CREB Signaling in Neurons | 1.98 | 0.12 | 0.426 | RAF1,GRIN2A,CAMK4,MAPK1,GRIN2D,GRIA2,GNB5,ITPR1,PRKCZ,GNAI3,PLCB4,PLCE1,GRIK5,CAMK2A,KL,ADCY5,PLCB1,ADCY8,ADCY10,MAP2K1,GRIA3,GRIK1,CAMK2G |
| Saline | P10-21 |  | 2.57 | 0.12 | 1.789 | GRIN2A,POLR2D,CAMK4,MAPK1,PDIA3,GRIN2D,GNB5,GNG13,ITPR1,PRKCZ,PRKCG,GNAI3,ADCY9,PLCB4,CAMK2A,GRIK4,ADCY5,PRKAR1B,PLCB1,ADCY8,MAP2K1,GRIA3,GRIK1 |
| VPA | P10-21 | CXCR4 Signaling | 2.21 | 0.127 | 0 | RAF1,MAPK1,EGR1,MAPK8,GNB5,ITPR1,PRKCZ,ROCK2,PTK2,ROCK1,GNAI3,PLCB4,RHOG,RND3,PAK3,KL,ADCY5,PLCB1,ADCY8,ADCY10,ELMO2,MAP2K1 |
| Saline | P10-21 |  | 2.46 | 0.121 | 1.886 | MAPK1,EGR1,GNB5,CXCL12,GNG13,ITPR1,PRKCZ,PRKCG,ROCK2,PTK2,ADCY9,GNAI3,PLCB4,RHOG,RND3,ADCY5,MAPK10,PLCB1,ADCY8,ELMO2,MAP2K1 |
| VPA | P10-21 | Cyclins and Cell Cycle Regulation | 2.93 | 0.179 | -1.897 | RAF1,SUV39H1,CCNB2,CCND1,CDK1,SKP2,CCNB1,CCNA2,MYT1,PPP2R3A,HDAC11,PPP2R2C,CDK2,CDC25A |
| Saline | P10-21 |  | 1.98 | 0.141 | -1.667 | MYT1,PPP2R3A,WEE1,SUV39H1,CDK4,CCNB2,PPP2R2C,CDK1,CDK2,SKP2,CCNB1 |
| VPA | P10-21 | D-myo-inositol-5-phosphate Metabolism | 2.42 | 0.135 |  | CDC25C,PPP1R1B,PIP4K2B,PAWR,PPP4R1,PPM1K,PPP1R14B,DUSP14,DUSP2,PLCH1,ATP1A1,PLCB4,PLCE1,PPP1R7,PPP2R3A,PLCB1,THTPA,PIP4K2C,PPP3CA,CDC25A,NUDT1 |
| Saline | P10-21 |  | 3.41 | 0.141 |  | CDC25C,PPFIBP2,PPP1R14A,PPP4R1,PPM1K,PPP1R14B,DUSP14,DUSP2,PLCH1,SET,ATP1A1,PLCB4,SYNJ1,DUSP1,PPP2R3A,PTPRO,PLCB1,PTPRN,THTPA,PIP4K2C,PPP3CA,NUDT1 |
| VPA | P10-21 | DNA damage-induced 14-3-3σ Signaling | 2.03 | 0.263 |  | CCNB2,CDK1,CDK2,CCNB1,RAD1 |
| Saline | P10-21 |  | 1.53 | 0.211 |  | CCNB2,CDK1,CDK2,CCNB1 |
| VPA | P10-21 | Dopamine Receptor Signaling | 1.9 | 0.143 | 0.632 | PPP1R14C,PPP1R1B,PPP1CB,PPP1R11,PPP1R14B,DRD1,PPP2R3A,PPP1R7,ADCY5,SLC18A1,PPP2R2C,ADCY10,ADCY8 |
| Saline | P10-21 |  | 1.52 | 0.121 | 0.707 | PPP1R14C,ADCY9,ADCY5,PPP2R3A,PRKAR1B,PPP1R14A,SLC18A1,PPP1R11,PPP2R2C,ADCY8,PPP1R14B |
| VPA | P10-21 | Dopamine-DARPP32 Feedback in cAMP Signaling | 5.12 | 0.17 | 1.279 | GRIN2A,CAMK4,KCNJ2,GRIN2D,PPP1CB,PRKG2,PPP1R14B,PRKCZ,GRINA,PLCE1,DRD1,PPP1R7,ADCY5,PPP3R1,PLCB1,PPP2R2C,ADCY8,PPP3CA,PPP1R14C,PPP1R1B,PPP1R11,PAWR,ITPR1,GRIN3A,GNAI3,PLCB4,PPP2R3A,CREM,CAMKK1,ADCY10 |
| Saline | P10-21 |  | 5.63 | 0.165 | 1.877 | GRIN2A,CAMK4,PDIA3,GRIN2D,PPP1R14B,PRKCZ,GRINA,KCNJ11,ADCY5,PPP3R1,PRKAR1B,PLCB1,PPP2R2C,ADCY8,PPP3CA,PPP1R14C,PPP1R14A,PPP1R11,ITPR1,PRKCG,GNAI3,ADCY9,PLCB4,KCNJ10,PPP2R3A,KCNJ9,CAMKK1,CREM,CAMKK2 |
| VPA | P10-21 | Endothelin-1 Signaling | 1.49 | 0.109 | -1.528 | PLA2G16,RAF1,MAPK1,EDNRB,CASP3,MAPK6,MAPK8,ITPR1,PRKCZ,MYC,GNAI3,PLCB4,PLCE1,EDN1,KL,ECE2,ADCY5,PLCB1,ADCY10,ADCY8,CASP7,PAFAH1B3 |
| Saline | P10-21 |  | 2.29 | 0.114 | 0.626 | PLA2G16,MAPK1,EDNRB,CASP3,MAPK4,PDIA3,MAPK6,ITPR1,PRKCZ,PRKCG,MYC,GNAI3,PLA2G6,ADCY9,PLCB4,EDN1,ADCY5,MAPK10,PLCB1,PTGS2,ADCY8,CASP7,PAFAH1B3 |
| VPA | P10-21 | Ephrin B Signaling | 2.17 | 0.16 | -1.265 | ROCK2,PTK2,ROCK1,VAV2,GNAI3,RAC2,MAPK1,GNB5,CAP1,EPHB3,EFNB3,LIMK1 |
| Saline | P10-21 |  | 1.69 | 0.133 | 0 | ROCK2,PTK2,VAV2,GNAI3,MAPK1,GNB5,CXCL12,CAP1,GNG13,EFNB3 |
| VPA | P10-21 | Epithelial Adherens Junction Signaling | 3.07 | 0.149 |  | VAV2,TUBB3,NOTCH3,LMO7,ACTN2,TUBB2A,AFDN,TCF7L1,TUBB,APC,TUBB2B,CTNNA2,MAGI1,TUBA1A,TUBB6,BAIAP2,MYH3,TUBA1C,TUBB4A,NOTCH1,ACVR1C,ACTN1 |
| Saline | P10-21 |  | 1.57 | 0.108 |  | VAV2,TUBB3,ACTN2,TUBB2A,TUBA4A,CTNNA1,TUBB,TUBB2B,CTNNA2,TUBA1A,MAGI1,TUBB6,BAIAP2,TUBB4A,TUBA1C,ACVR1C |
| VPA | P10-21 | ERK/MAPK Signaling | 1.47 | 0.108 | -0.426 | ITGB1,MYCN,PPP1R14C,RAF1,RAC2,PTK2B,MAPK1,PPP1CB,PPP1R11,MKNK2,PPP1R14B,DUSP2,PTK2,MYC,PAK3,PPP1R7,KL,ETS2,PPP2R3A,MKNK1,PPP2R2C,MAP2K1 |
| Saline | P10-21 |  | 1.45 | 0.0985 | 0.894 | MYCN,PPP1R14C,PTK2B,MAPK1,CRKL,PPP1R14A,PPP1R11,PPP1R14B,DUSP2,PRKCG,MYC,PTK2,PLA2G6,H3F3A/H3F3B,DUSP1,ETS2,PPP2R3A,PRKAR1B,PPP2R2C,MAP2K1 |
| VPA | P10-21 | Estrogen-mediated S-phase Entry | 2.96 | 0.292 | -2.646 | MYC,CCNA2,CCND1,CDK1,CDK2,SKP2,CDC25A |
| Saline | P10-21 |  | 1.79 | 0.208 | -2.236 | MYC,CDK4,CDK1,CDK2,SKP2 |
| VPA | P10-21 | Fatty Acid Activation | 2.8 | 0.316 |  | ACSL3,SLC27A5,SLC27A2,ACSL6,ACSL4,SLC27A3 |
| Saline | P10-21 |  | 1.53 | 0.211 |  | ACSL3,SLC27A5,SLC27A2,ACSL6 |
| VPA | P10-21 | G-Protein Coupled Receptor Signaling | 4.04 | 0.137 |  | ADRA2B,RAF1,GPR17,CAMK4,HTR4,MAPK1,PTK2B,NFKBIE,PDE1A,HRH3,CAMK2A,DRD1,ADCY5,KL,PLCB1,SMPDL3B,RGS14,ADCY8,MAP2K1,HCAR2,HTR6,RGS2,NPY1R,MC4R,PDE1C,GNAI3,GABBR2,PLCB4,LPAR1,RASGRP1,PDE1B,S1PR1,PDE8B,HTR1F,ADRA2C,ADCY10,ADORA2A,CAMK2G |
| Saline | P10-21 |  | 2.19 | 0.104 |  | CAMK4,MAPK1,PTK2B,CHRM4,PDPK1,PDE1A,HRH3,AVPR1A,CAMK2A,ADCY5,HTR7,PRKAR1B,PLCB1,ADCY8,MAP2K1,ADRB2,PDE2A,RGS4,PRKCG,GNAI3,ADCY9,PLCB4,TULP2,LPAR1,DUSP1,RASGRP1,PDE1B,S1PR1,CALCR |
| VPA | P10-21 | GABA Receptor Signaling | 1.84 | 0.151 |  | SLC6A11,GABBR2,GABRR2,GPR37,GABRA4,UBQLN1,ADCY5,GAD1,ADCY8,GABRD,ADCY10 |
| Saline | P10-21 |  | 2.2 | 0.151 |  | SLC6A11,NSF,ADCY9,GPR37,GABRA4,ADCY5,AP2B1,ADCY8,GABRD,GABRA1,AP2A2 |
| VPA | P10-21 | GADD45 Signaling | 1.37 | 0.211 |  | CCND1,CDK1,CDK2,CCNB1 |
| Saline | P10-21 |  | 1.53 | 0.211 |  | CDK4,CDK1,CDK2,CCNB1 |
| VPA | P10-21 | Gap Junction Signaling | 4.04 | 0.155 |  | RAF1,MAPK1,PRKG2,TUBB,PRKCZ,TUBB2B,PLCE1,SP1,DRD1,ADCY5,KL,PPP3R1,PLCB1,TUBA1C,TUBB4A,ADCY8,MAP2K1,PPP3CA,TUBB3,TUBB2A,ITPR1,GNAI3,PLCB4,TUBA1A,LPAR1,TUBB6,SGSM3,ADCY10 |
| Saline | P10-21 |  | 4.06 | 0.144 |  | TUBB3,MAPK1,PDIA3,TUBB2A,TUBA4A,ITPR1,TUBB,PRKCZ,TUBB2B,PRKCG,ADCY9,GNAI3,PLCB4,TUBA1A,LPAR1,TUBB6,ADCY5,PPP3R1,SGSM3,PRKAR1B,PLCB1,TUBA1C,TUBB4A,ADCY8,MAP2K1,PPP3CA |
| VPA | P10-21 | Germ Cell-Sertoli Cell Junction Signaling | 3.47 | 0.148 |  | RAC2,MAPK1,AFDN,TUBB,TUBB2B,LIMK1,PTK2,MAP3K10,CTNNA2,RHOG,KL,TUBA1C,TUBB4A,MAP2K1,ACTN1,ITGB1,MAP3K9,TUBB3,ACTN2,TUBB2A,MAPK8,GSN,TUBA1A,RND3,TUBB6,PAK3 |
| Saline | P10-21 |  | 2.05 | 0.114 |  | TUBB3,MAPK1,ACTN2,TUBB2A,TUBA4A,CTNNA1,PDPK1,TUBB,GSN,TUBB2B,PTK2,CTNNA2,RHOG,TUBA1A,TUBB6,RND3,MAPK10,TUBA1C,TUBB4A,MAP2K1 |
| VPA | P10-21 | Glutamate Receptor Signaling | 5.1 | 0.25 | 0 | GRIN2A,CAMK4,SLC17A6,GRIN2D,GRIA2,SLC1A3,GRIP1,GRIN3A,GRINA,GRIK5,PICK1,SLC1A2,GLUL,HOMER1,GRIK1,GRIA3 |
| Saline | P10-21 |  | 3.77 | 0.203 | -0.378 | SLC1A6,GRIN2A,CAMK4,GRIN2D,SLC1A3,GRIP1,GRINA,GRIK4,GLUL,SLC1A2,HOMER1,GRIK1,GRIA3 |
| VPA | P10-21 | Glycolysis I | 2.11 | 0.195 |  | PKLR,PGAM1,PKM,Tpi1 (includes others),PFKM,ALDOC,FBP2,NAD+ |
| Saline | P10-21 |  | 3.71 | 0.244 |  | PGK1,GPI,PKLR,PGAM1,PKM,ALDOA,PFKL,Tpi1 (includes others),PFKM,ALDOC |
| VPA | P10-21 | GNRH Signaling | 2.46 | 0.141 | 0.229 | RAF1,MAP3K9,MAPK1,EGR1,MAPK8,ITPR1,PRKCZ,PTK2,MAP3K10,GNAI3,PLCB4,CAMK2A,PAK3,ADCY5,PLCB1,ADCY10,ADCY8,MAP2K1,CAMK2G |
| Saline | P10-21 |  | 1.91 | 0.119 | 2.5 | MAPK1,EGR1,ITPR1,PRKCZ,PRKCG,PTK2,ADCY9,GNAI3,PLCB4,CAMK2A,ADCY5,PRKAR1B,MAPK10,PLCB1,ADCY8,MAP2K1 |
| VPA | P10-21 | Gα12/13 Signaling | 2.25 | 0.137 | 0.243 | VAV2,RAF1,PTK2B,MAPK1,F2R,NFKBIE,MAPK8,CDH19,ROCK2,PTK2,ROCK1,TEC,LPAR1,LPAR2,KL,CDH8,MAP2K1,CDH13 |
| Saline | P10-21 |  | 1.4 | 0.107 | 1.604 | PTK2,ROCK2,VAV2,F2RL2,CDH9,LPAR2,LPAR1,PTK2B,MAPK1,CDH3,MEF2D,MAPK10,MAP2K1,CDH19 |
| VPA | P10-21 | Gαq Signaling | 1.76 | 0.119 | 0.943 | RAF1,RGS2,CAMK4,PTK2B,MAPK1,NFKBIE,CSK,GNB5,ITPR1,PRKCZ,ROCK2,ROCK1,PLCB4,RHOG,RND3,KL,PPP3R1,PLCB1,MAP2K1,PPP3CA |
| Saline | P10-21 |  | 2.27 | 0.119 | 1.886 | CAMK4,PTK2B,MAPK1,GNB5,GNG13,RGS4,ITPR1,AVPR1A,PRKCZ,PRKCG,ROCK2,PLCB4,RHOG,RND3,PPP3R1,CALCR,PLCB1,ARHGEF25,MAP2K1,PPP3CA |
| VPA | P10-21 | Gαs Signaling | 1.83 | 0.133 | 1.604 | HTR6,RGS2,ADD2,MAPK1,HTR4,GNB5,MC4R,DRD1,ADCY5,ADD1,ADCY8,ADCY10,ADORA2A,MAP2K1,HCAR2 |
| Saline | P10-21 |  | 1.89 | 0.124 | 0.905 | ADD2,MAPK1,RYR2,GNB5,GNG13,CNGA1,ADCY9,ADCY5,HTR7,ADD1,PRKAR1B,ADCY8,MAP2K1,ADRB2 |
| VPA | P10-21 | Huntington's Disease Signaling | 1.97 | 0.113 | -1.964 | MAPK1,GNB5,PRKCZ,MAP3K10,ARFIP2,IGF1,SP1,KL,HDAC11,PLCB1,NAPA,CAPN5,ATP5J,CAPN6,CASP3,MAPK8,APAF1,ITPR1,HIP1,SNAP25,BCL2L1,PLCB4,PSME1,CACNA1B,PENK,STX16,CAPN2,CASP7 |
| Saline | P10-21 |  | 2.07 | 0.105 | -1 | POLR2D,MAPK1,PACSIN1,GNB5,PDPK1,GNG13,AP2A2,PRKCZ,NSF,IGF1,PLCB1,NAPA,CAPN6,CASP3,PSME2,APAF1,BAX,ITPR1,SNAP25,RPH3A,PRKCG,BCL2L1,PLCB4,PSME1,STX16,CASP7 |
| VPA | P10-21 | Leukocyte Extravasation Signaling | 3.1 | 0.134 | 0.426 | RAC2,MAPK1,PTK2B,MMP14,Wasl,MMP15,AFDN,PRKCZ,ROCK2,PTK2,CTNNA2,KL,TIMP1,ARHGAP12,CLDN9,ACTN1,VAV2,ITGB1,CLDN10,ACTN2,MAPK8,THY1,SELPLG,TEC,ROCK1,GNAI3,JAM3,RASGRP1,ARHGAP35 |
| Saline | P10-21 |  | 1.44 | 0.0972 | 2.183 | VAV2,CLDN10,PTK2B,MAPK1,Wasl,CRKL,ACTN2,CTNNA1,CXCL12,THY1,PRKCZ,SELPLG,PRKCG,PTK2,ROCK2,GNAI3,CTNNA2,TIMP1,RASGRP1,MAPK10,CLDN3 |
| VPA | P10-21 | LPS/IL-1 Mediated Inhibition of RXR Function | 1.95 | 0.115 | 0.816 | ECSIT,NDST3,CYP3A7,SLC27A2,CPT1B,SLC27A5,ALDH1A1,ACSL4,FABP5,FABP7,HS3ST1,HMGCS1,IL1RAP,TNFRSF11B,GSTA3,GSTA2,ABCB1,ACSL3,ACSL6,MAPK8,Sult1d1,IL33,CHST1,IL1RN,ALDH3B1,SLC27A3 |
| Saline | P10-21 |  | 1.49 | 0.0973 | 0.447 | ECSIT,GSTA3,CYP3A7,ABCB1,NDST3,GSTA2,ACSL3,SLC27A2,ACSL6,Sult1d1,CHST1,SLC27A5,ALDH1A1,SCARB1,ALDH1L2,IL1RN,FABP7,ALDH3B1,FMO1,FABP5,CPT1C,TNFRSF11B |
| VPA | P10-21 | Melatonin Signaling | 1.68 | 0.143 | 0.632 | GNAI3,RAF1,PLCB4,CAMK4,PLCE1,CAMK2A,MAPK1,PLCB1,MAP2K1,PRKCZ,CAMK2G |
| Saline | P10-21 |  | 2.02 | 0.143 | 1.508 | GNAI3,PLCB4,CAMK4,CAMK2A,MAPK1,PDIA3,PRKAR1B,PLCB1,MAP2K1,PRKCZ,PRKCG |
| VPA | P10-21 | Mitochondrial L-carnitine Shuttle Pathway | 3.35 | 0.333 |  | ACSL3,SLC27A5,SLC27A2,CPT1B,ACSL6,ACSL4,SLC27A3 |
| Saline | P10-21 |  | 2.04 | 0.238 |  | ACSL3,SLC27A5,SLC27A2,ACSL6,CPT1C |
| VPA | P10-21 | Mitotic Roles of Polo-Like Kinase | 7.74 | 0.303 | -2 | FZR1,KIF23,CDC25C,ESPL1,CDC20,PLK3,PTTG1,PRC1,CCNB2,ANAPC1,CDK1,CCNB1,PPP2R3A,PLK2,PKMYT1,FBXO5,PPP2R2C,CDC16,KIF11,CDC25A |
| Saline | P10-21 |  | 5.54 | 0.242 | -1.941 | KIF23,CDC25C,CDC20,PLK3,WEE1,PTTG1,PRC1,CCNB2,PLK1,CDK1,CCNB1,PPP2R3A,PLK2,FBXO5,PPP2R2C,KIF11 |
| VPA | P10-21 | Neuropathic Pain Signaling In Dorsal Horn Neurons | 2.36 | 0.144 | 1.213 | GRIN2A,CAMK4,MAPK1,GRIN2D,GRIA2,ITPR1,PRKCZ,GRINA,GRIN3A,PLCB4,CAMK2A,PLCE1,GPR37,KL,PLCB1,CAMK2G,GRIA3 |
| Saline | P10-21 |  | 2.09 | 0.127 | 2.84 | GRIN2A,CAMK4,MAPK1,PDIA3,GRIN2D,ITPR1,PRKCZ,GRINA,PRKCG,PLCB4,CAMK2A,GPR37,PRKAR1B,PLCB1,GRIA3 |
| VPA | P10-21 | nNOS Signaling in Neurons | 4.94 | 0.269 | 1.667 | CAPN5,CAPN6,GRIN2A,CAMK4,GRIN2D,RASD1,PRKCZ,GRINA,PFKM,GRIN3A,CAMK2A,PPP3R1,CAPN2,PPP3CA |
| Saline | P10-21 |  | 4.08 | 0.231 | 2.121 | CAPN6,GRIN2A,CAMK4,CAMK2A,GRIN2D,PPP3R1,RASD1,PRKCZ,PPP3CA,GRINA,PFKM,PRKCG |
| VPA | P10-21 | Parkinson's Signaling | 2.38 | 0.312 |  | CASP3,MAPK1,GPR37,MAPK8,SNCAIP |
| Saline | P10-21 |  | 2.6 | 0.312 |  | SEPT5,CASP3,MAPK1,GPR37,SNCAIP |
| VPA | P10-21 | phagosome maturation | 3.33 | 0.152 |  | CTSK,TUBB3,ATP6V1D,VPS41,PRDX5,TUBB2A,DYNLT1,TUBB,SNAP25,PRDX6,TUBB2B,DYNC1H1,TUBA1A,Dync1i2,TUBB6,CTSS,Atp6ap1l,STX16,TUBA1C,TUBB4A,ATP6V1G2,NAPA,ATP6V0E1 |
| Saline | P10-21 |  | 4.5 | 0.159 |  | CTSK,VPS18,TUBB3,VPS41,PRDX1,TUBB2A,TUBA4A,DYNLT1,TUBB,ATP6V1A,SNAP25,PRDX6,TUBB2B,NSF,CTSA,TUBA1A,Dync1i2,TUBB6,CTSS,STX16,TUBA1C,TUBB4A,NAPA,ATP6V1B2 |
| VPA | P10-21 | PI3K Signaling in B Lymphocytes | 2.18 | 0.135 |  | VAV2,CD81,RAF1,CAMK4,MAPK1,NFKBIE,ITPR1,FCGR2B,PRKCZ,PLCB4,CAMK2A,PLCE1,PPP3R1,SH2B2,PLCB1,MAP2K1,PPP3CA,CAMK2G |
| Saline | P10-21 |  | 1.97 | 0.12 |  | VAV2,CD81,CAMK4,MAPK1,PDIA3,PDPK1,ATF6,ITPR1,PRKCZ,PLCB4,CAMK2A,PPP3R1,SH2B2,PLCB1,MAP2K1,PPP3CA |
| VPA | P10-21 | Production of Nitric Oxide and Reactive Oxygen Species in Macrophages | 1.81 | 0.116 | 0.626 | MAP3K9,PPP1R14C,MAPK1,NFKBIE,MAPK8,PPP1CB,PPP1R11,PPP1R14B,PRKCZ,MAP3K10,ALB,LYZ,RHOG,RND3,PPP1R7,KL,PPP2R3A,PPP2R2C,MAP2K1,CLU,TNFRSF11B,RBP4,APOD |
| Saline | P10-21 |  | 1.53 | 0.101 | 2.683 | PPP1R14C,MAPK1,PPP1R14A,ARG2,PPP1R11,IFNGR1,PPP1R14B,PRKCZ,PRKCG,ALB,LYZ,RHOG,RND3,PPP2R3A,MAPK10,PPP2R2C,MAP2K1,TNFRSF11B,RBP4,APOD |
| VPA | P10-21 | Protein Kinase A Signaling | 6.25 | 0.142 | 3.286 | RAF1,MAPK1,GNB5,PPP1R14B,PTK2,ROCK2,CAMK2A,PHKB,DUSP3,PLCE1,PPP1R7,ADCY5,PPP3R1,PLCB1,SMPDL3B,CDC16,CDC25A,PPP1R14C,ADD2,PPP1R1B,PTCH1,PPP1R11,ITPR1,PDE1C,PYGM,PDE1B,CREM,H1f0,ADCY10,EBI3,CAMK2G,AKAP12,CAMK4,AKAP8,PTK2B,NFKBIE,PPP1CB,PDE1A,ANAPC1,PRKCZ,DUSP2,FLNA,PTPRZ1,ADCY8,MAP2K1,PPP3CA,AKAP5,CDC25C,Cdkn3,TCF7L1,ROCK1,GNAI3,PLCB4,CDC14B,ADD1,PDE8B,Ptprt |
| Saline | P10-21 |  | 6.37 | 0.132 | 3.727 | MAPK1,GNB5,GNG13,PPP1R14B,PTK2,ROCK2,DUSP3,CAMK2A,PTPRO,ADCY5,PPP3R1,PLCB1,PPP1R14C,PDE2A,ADD2,PPP1R14A,PPP1R11,ITPR1,CNGA1,ADCY9,TULP2,PYGM,H3F3A/H3F3B,DUSP1,CREM,PDE1B,H1f0,EBI3,AKAP12,CAMK4,AKAP8,PTK2B,PDIA3,UBASH3B,PDE1A,PRKCZ,DUSP2,AKAP11,PRKAR1B,PTPRZ1,ADCY8,MAP2K1,PTPRN,PPP3CA,AKAP5,CDC25C,RYR2,Cdkn3,PRKCG,GNAI3,PLCB4,ADD1,PTGS2 |
| VPA | P10-21 | PTEN Signaling | 1.59 | 0.124 | 0.258 | MAST2,ITGB1,RAF1,RAC2,MAPK1,CASP3,PREX2,CCND1,PRKCZ,PTK2,BCL2L1,MAGI1,PDGFRA,MAP2K1,MAGI3 |
| Saline | P10-21 |  | 1.34 | 0.107 | -0.832 | PTK2,SYNJ2,MAST2,BCL2L1,MAGI1,SYNJ1,CASP3,MAPK1,PDGFRA,PDPK1,PREX2,MAP2K1,PRKCZ |
| VPA | P10-21 | Regulation of Cellular Mechanics by Calpain Protease | 3.15 | 0.207 | 0.632 | ITGB1,PTK2,CAPN5,CCNA2,CAPN6,MAPK1,ACTN2,CAPN2,CCND1,CDK1,ACTN1,CDK2 |
| Saline | P10-21 |  | 1.52 | 0.138 | 1.342 | PTK2,CAPN6,MAPK1,ACTN2,CDK4,CNGA1,CDK1,CDK2 |
| VPA | P10-21 | Regulation of eIF4 and p70S6K Signaling | 1.67 | 0.118 | 0 | ITGB1,RAF1,EIF4EBP2,MAPK1,RPS18,RPS29,PRKCZ,RPS4Y1,EIF4G2,PPP2R3A,KL,PAIP1,MKNK1,RPS27L,EIF4A1,PPP2R2C,RPS2,MAP2K1,RPSA |
| Saline | P10-21 |  | 5.93 | 0.174 | 1.633 | MAPK1,RPS27,RPS18,PDPK1,PRKCZ,RPS28,RPS7,RPS20,PAIP1,EIF3A,PPP2R2C,RPS2,RPS12,RPS5,MAP2K1,RPS24,RPS8,RPS10,RPS29,RPS15,RPS4Y1,RPS16,RPS26,PPP2R3A,EIF4A1,RPS27L,RPS15A,RPSA |
| VPA | P10-21 | Remodeling of Epithelial Adherens Junctions | 3.56 | 0.206 |  | TUBB3,RALA,MAPRE1,ACTN2,TUBB2A,TUBB,APC,TUBB2B,CTNNA2,TUBA1A,TUBB6,TUBB4A,TUBA1C,ACTN1 |
| Saline | P10-21 |  | 3.49 | 0.191 |  | CTNNA2,TUBB3,TUBA1A,TUBB6,MAPRE1,ACTN2,TUBB2A,TUBA4A,CTNNA1,TUBA1C,TUBB4A,TUBB,TUBB2B |
| VPA | P10-21 | Role of CHK Proteins in Cell Cycle Checkpoint Control | 1.39 | 0.145 | 0 | CDC25C,PPP2R3A,PPP2R2C,CDK1,CDK2,CDC25A,RFC3,RAD1 |
| Saline | P10-21 |  | 1.64 | 0.145 | 0.378 | CDC25C,PPP2R3A,PPP2R2C,PLK1,CDK1,CDK2,CHEK1,RFC3 |
| VPA | P10-21 | Role of NFAT in Cardiac Hypertrophy | 2.37 | 0.126 | 0 | AKAP5,RAF1,CAMK4,MAPK1,SLC8A3,MAPK8,GNB5,ITPR1,PRKCZ,GNAI3,PLCB4,PLCE1,CAMK2A,IGF1,KL,ADCY5,PPP3R1,HDAC11,PLCB1,ADCY10,SLC8A1,ADCY8,MAP2K1,PPP3CA,CAMK2G |
| Saline | P10-21 |  | 2.37 | 0.116 | 2.837 | AKAP5,CAMK4,MAPK1,PDIA3,GNB5,GNG13,ITPR1,PRKCZ,PRKCG,ADCY9,GNAI3,PLCB4,CAMK2A,IGF1,ADCY5,PPP3R1,MEF2D,MAPK10,PRKAR1B,PLCB1,ADCY8,MAP2K1,PPP3CA |
| VPA | P10-21 | Semaphorin Signaling in Neurons | 4.83 | 0.264 |  | DPYSL2,ITGB1,MAPK1,DPYSL3,DPYSL5,LIMK1,ROCK2,PTK2,ROCK1,CRMP1,RHOG,RND3,PAK3,NRP1 |
| Saline | P10-21 |  | 2.23 | 0.17 |  | ROCK2,PTK2,CRMP1,SEMA4D,RHOG,RND3,MAPK1,DPYSL3,DPYSL5 |
| VPA | P10-21 | Sertoli Cell-Sertoli Cell Junction Signaling | 3.1 | 0.14 |  | SPTBN2,ITGB1,MAP3K9,RAF1,CLDN10,DLG1,TUBB3,MAPK1,ACTN2,TUBB2A,MAPK8,AFDN,PRKG2,TUBB,TUBB2B,MAP3K10,CTNNA2,TUBA1A,TUBB6,JAM3,TUBA1C,TUBB4A,CLDN9,ADCY10,MAP2K1,ACTN1 |
| Saline | P10-21 |  | 1.81 | 0.108 |  | SPTBN2,DLG1,CLDN10,TUBB3,MAPK1,ACTN2,TUBB2A,CTNNA1,TUBA4A,TUBB,TUBB2B,CTNNA2,TUBA1A,TUBB6,PRKAR1B,MAPK10,TUBA1C,TUBB4A,MAP2K1,CLDN3 |
| VPA | P10-21 | Signaling by Rho Family GTPases | 3.03 | 0.129 | -0.557 | RAF1,SEPT9,MAPK1,PTK2B,SEPT3,ARHGEF7,GNB5,PIP4K2B,PRKCZ,LIMK1,ROCK2,STMN1,PTK2,MAP3K10,RHOG,ARFIP2,KL,BAIAP2,ARHGEF3,CDH13,MAP2K1,ITGB1,MAP3K9,MAPK8,CDH19,ROCK1,GNAI3,RND3,PAK3,CDH8,ARHGEF9,PIP4K2C |
| Saline | P10-21 |  | 1.32 | 0.0924 | 3.441 | SEPT5,PTK2B,MAPK1,GNB5,GNG13,SEPT11,PRKCZ,CDH19,STMN1,ROCK2,PTK2,GNAI3,PIP5K1A,CDH9,RHOG,RND3,CDH3,BAIAP2,MAPK10,ARHGEF3,ARHGEF9,MAP2K1,PIP4K2C |
| VPA | P10-21 | Sperm Motility | 1.37 | 0.114 | 2.324 | PLA2G16,CACNA1G,CAMK4,PTK2B,PRKG2,ITPR1,PDE1A,PRKCZ,PDE1C,PTK2,PLCB4,PLCE1,PDE1B,PLCB1,ADCY10,PAFAH1B3 |
| Saline | P10-21 |  | 2.1 | 0.121 | 3.153 | PLA2G16,PDE2A,CAMK4,PTK2B,PDIA3,ITPR1,PDE1A,CNGA1,PRKCZ,PRKCG,PTK2,PLA2G6,PLCB4,PDE1B,PRKAR1B,PLCB1,PAFAH1B3 |
| VPA | P10-21 | Sphingosine-1-phosphate Signaling | 2.43 | 0.143 | 0 | PTK2B,MAPK1,CASP3,PDGFA,PTK2,GNAI3,PLCB4,RHOG,PLCE1,RND3,ADCY5,KL,PDGFRA,S1PR1,PLCB1,ADCY10,ADCY8,CASP7 |
| Saline | P10-21 |  | 2.19 | 0.127 | 1 | PTK2B,MAPK1,CASP3,PDIA3,PTK2,GNAI3,ADCY9,PLCB4,RHOG,RND3,ADCY5,PDGFRA,S1PR1,PLCB1,ADCY8,CASP7 |
| VPA | P10-21 | Synaptic Long Term Depression | 1.54 | 0.116 | 0.471 | PLA2G16,RAF1,MAPK1,GRIA2,PPP1R17,PRKG2,ITPR1,PRKCZ,GNAI3,PLCB4,PLCE1,IGF1,PPP2R3A,PLCB1,PPP2R2C,MAP2K1,GRIA3,PAFAH1B3 |
| Saline | P10-21 |  | 1.69 | 0.11 | 1.698 | PLA2G16,MAPK1,PDIA3,RYR2,ITPR1,PRKCZ,PRKCG,PLA2G6,GNAI3,PLCB4,IGF1,PPP2R3A,PLCB1,PPP2R2C,MAP2K1,GRIA3,PAFAH1B3 |
| VPA | P10-21 | Synaptic Long Term Potentiation | 5.44 | 0.195 | 0.816 | RAF1,GRIN2A,CAMK4,MAPK1,GRIN2D,PPP1CB,PPP1R14B,PRKCZ,GRINA,CAMK2A,PLCE1,PPP1R7,PPP3R1,PLCB1,ADCY8,MAP2K1,PPP3CA,PPP1R14C,GRIA2,PPP1R11,ITPR1,GRIN3A,PLCB4,GRIA3,CAMK2G |
| Saline | P10-21 |  | 4.72 | 0.172 | 2.4 | PPP1R14C,GRIN2A,CAMK4,MAPK1,PDIA3,GRIN2D,PPP1R14A,PPP1R11,ITPR1,PPP1R14B,PRKCZ,PRKCG,GRINA,PLCB4,CAMK2A,PPP3R1,PRKAR1B,PLCB1,ADCY8,MAP2K1,PPP3CA,GRIA3 |
| VPA | P10-21 | Thrombin Signaling | 2.38 | 0.124 | 0.2 | RAF1,CAMK4,MAPK1,F2R,GNB5,PPP1CB,PRKCZ,ROCK2,PTK2,RHOG,PLCE1,CAMK2A,KL,ADCY5,PLCB1,ARHGEF3,ADCY8,MAP2K1,ITPR1,ROCK1,GNAI3,PLCB4,RND3,ADCY10,ARHGEF9,CAMK2G |
| Saline | P10-21 |  | 2.41 | 0.115 | 2.558 | F2RL2,CAMK4,MAPK1,PDIA3,GNB5,GNG13,PDPK1,ITPR1,PRKCZ,PRKCG,ROCK2,PTK2,GNAI3,ADCY9,PLCB4,RHOG,CAMK2A,RND3,ADCY5,PLCB1,ARHGEF3,ADCY8,ARHGEF9,MAP2K1 |
| VPA | P10-21 | α-Adrenergic Signaling | 2.44 | 0.155 | 1.069 | RAF1,CAMK4,MAPK1,SLC8A3,GNB5,ITPR1,PRKCZ,GNAI3,PYGM,PHKB,ADCY5,ADCY10,SLC8A1,ADCY8,MAP2K1 |
| Saline | P10-21 |  | 2.47 | 0.144 | 1.732 | GNAI3,ADCY9,CAMK4,PYGM,MAPK1,ADCY5,GNB5,PRKAR1B,GNG13,ITPR1,ADCY8,MAP2K1,PRKCZ,PRKCG |

Table S1B.

| *Group* | *Comparison* | *Ingenuity Canonical Pathways* | *-log(p-value)* | *Ratio* | *z-score* | *Molecules* |
| --- | --- | --- | --- | --- | --- | --- |
| Saline | P10-21 | 3-phosphoinositide Biosynthesis | 2.07 | 0.111 |  | CDC25C,PPFIBP2,PPP1R14A,ERBB3,PPP4R1,PPM1K,PPP1R14B,DUSP14,DUSP2,SET,PIP5K1A,ATP1A1,SYNJ1,DUSP1,PPP2R3A,PTPRO,PDGFRA,PTPRN,THTPA,PIP4K2C,PPP3CA,NUDT1 |
| Saline | P10-21 | 3-phosphoinositide Degradation | 2.32 | 0.123 |  | CDC25C,PPFIBP2,PPP1R14A,PPP4R1,PPM1K,PPP1R14B,DUSP14,DUSP2,SYNJ2,SET,ATP1A1,SYNJ1,DUSP1,PPP2R3A,PTPRO,PTPRN,THTPA,PPP3CA,NUDT1 |
| Saline | P10-21 | ATM Signaling | 1.51 | 0.125 | 1.414 | CDC25C,SUV39H1,MAPK10,CCNB2,HIST1H4J,CBX5,CDK1,CDK2,CHEK1,CCNB1 |
| Saline | P10-21 | Calcium-induced T Lymphocyte Apoptosis | 1.48 | 0.129 | 2.333 | CAMK4,PPP3R1,MEF2D,NR4A1,HLA-DOB,ITPR1,PRKCZ,PPP3CA,PRKCG |
| Saline | P10-21 | CCR5 Signaling in Macrophages | 1.34 | 0.122 |  | GNAI3,CAMK4,MAPK1,PTK2B,GNB5,MAPK10,GNG13,PRKCZ,PRKCG |
| Saline | P10-21 | Corticotropin Releasing Hormone Signaling | 1.99 | 0.124 | 1.941 | CAMK4,MAPK1,UCN3,ITPR1,PRKCZ,PRKCG,GNAI3,ADCY9,ADCY5,MEF2D,PRKAR1B,NR4A1,PTGS2,ADCY8,MAP2K1 |
| Saline | P10-21 | D-myo-inositol (1,4,5,6)-Tetrakisphosphate Biosynthesis | 2.55 | 0.131 |  | CDC25C,PPFIBP2,PPP1R14A,PPP4R1,PPM1K,PPP1R14B,DUSP14,DUSP2,SET,ATP1A1,SYNJ1,DUSP1,PPP2R3A,PTPRO,PTPRN,PPP3CA,THTPA,NUDT1 |
| Saline | P10-21 | D-myo-inositol (3,4,5,6)-tetrakisphosphate Biosynthesis | 2.55 | 0.131 |  | CDC25C,PPFIBP2,PPP1R14A,PPP4R1,PPM1K,PPP1R14B,DUSP14,DUSP2,SET,ATP1A1,SYNJ1,DUSP1,PPP2R3A,PTPRO,PTPRN,PPP3CA,THTPA,NUDT1 |
| Saline | P10-21 | EIF2 Signaling | 13.4 | 0.228 | -3.545 | RPL24,RPL11,MAPK1,RPS27,Rpl36a,RPS18,RPL39,PDPK1,RPL7,RPS7,RPS28,RPL27A,RPL14,RPL35,RPS20,RPL18A,PAIP1,EIF3A,RPS2,RPS12,RPL36,RPS5,MAP2K1,RPL31,RPL18,RPS24,RPS8,RPS10,RPL12,RPS29,RPL10A,RPL15,RPL27,RPS4Y1,RPS15,RPS16,RPS26,RPS27L,EIF4A1,RPL10,RPS15A,RPL6,RPL41,RPSA,RPLP0 |
| Saline | P10-21 | Fatty Acid α-oxidation | 1.72 | 0.2 |  | ALDH1A1,ALDH1L2,ALDH3B1,PTGS2,ALOXE3 |
| Saline | P10-21 | GPCR-Mediated Integration of Enteroendocrine Signaling Exemplified by an L Cell | 1.9 | 0.138 |  | GNAI3,ADCY9,PLCB4,GIPR,PDIA3,ADCY5,PRKAR1B,PLCB1,ITPR1,ADCY8,ADRB2 |
| Saline | P10-21 | GPCR-Mediated Nutrient Sensing in Enteroendocrine Cells | 1.86 | 0.13 |  | GNAI3,ADCY9,PLCB4,PDIA3,ADCY5,PRKAR1B,PLCB1,GNG13,ITPR1,ADCY8,PRKCZ,PRKCG |
| Saline | P10-21 | Hypusine Biosynthesis | 1.45 | 0.4 |  | DHPS,DOHH |
| Saline | P10-21 | mTOR Signaling | 7.55 | 0.176 | 1.069 | MAPK1,PRKAB2,RPS27,RPS18,PDPK1,FKBP1A,PRKCZ,RPS7,RPS28,RHOG,RPS20,EIF3A,PPP2R2C,RPS2,RPS12,RPS5,RPS24,RHEB,RPS8,RPS10,RPS29,PRKCG,ATG13,DGKZ,RPS6KA6,RPS4Y1,RPS15,RPS16,RND3,PPP2R3A,RPS26,RPS27L,EIF4A1,PRR5,RPS15A,RPSA |
| Saline | P10-21 | Netrin Signaling | 1.69 | 0.159 |  | PPP3R1,ABLIM3,RYR2,UNC5D,PRKAR1B,ABLIM2,PPP3CA |
| Saline | P10-21 | Nur77 Signaling in T Lymphocytes | 1.44 | 0.133 |  | CAMK4,CASP3,PPP3R1,MEF2D,APAF1,NR4A1,HLA-DOB,PPP3CA |
| Saline | P10-21 | P2Y Purigenic Receptor Signaling Pathway | 1.42 | 0.106 | 1.941 | MAPK1,PDIA3,GNB5,GNG13,PRKCZ,PRKCG,MYC,GNAI3,ADCY9,PLCB4,ADCY5,PRKAR1B,PLCB1,ADCY8,MAP2K1 |
| Saline | P10-21 | Protein Ubiquitination Pathway | 1.59 | 0.0965 |  | USP24,CRYAB,USP45,CDC20,DNAJB4,USP20,USP2,USP8,USP42,DNAJC16,HSPB6,HSPA4L,DNAJC9,DNAJC27,HSPH1,PSME2,SKP2,USP31,PSME1,USP22,USP29,BAP1,USP34,DNAJB5,UBE2C |
| Saline | P10-21 | RAR Activation | 2.75 | 0.122 |  | DHRS3,MAPK1,AKR1C3,CYP26A1,SMARCE1,PDPK1,RBP1,CRABP1,PRKCZ,SMARCD3,PRKCG,ADCY9,LRAT,TNIP1,ALDH1A1,DUSP1,ADCY5,MAPK10,PRKAR1B,GTF2H5,RDH12,ADCY8,MAP2K1,RBP4 |
| Saline | P10-21 | Retinoate Biosynthesis I | 1.5 | 0.158 |  | DHRS3,Akr1b10,ALDH1A1,AKR1C3,RDH12,RBP1 |
| Saline | P10-21 | Role of MAPK Signaling in the Pathogenesis of Influenza | 1.31 | 0.12 |  | PLA2G16,PLA2G6,CASP3,MAPK1,MAPK10,BAX,PTGS2,MAP2K1,PAFAH1B3 |
| Saline | P10-21 | Superpathway of Inositol Phosphate Compounds | 2.3 | 0.108 |  | PPFIBP2,PPM1K,PPP1R14B,DUSP2,PLCH1,SET,ATP1A1,PTPRO,PDGFRA,PLCB1,PTPRN,PPP3CA,CDC25C,PPP1R14A,ERBB3,ITPKA,PPP4R1,DUSP14,SYNJ2,PLCB4,PIP5K1A,SYNJ1,DUSP1,PPP2R3A,PIP4K2C,THTPA,NUDT1 |
| Saline | P10-21 | The Visual Cycle | 2.46 | 0.219 |  | DHRS3,LRAT,Akr1b10,AKR1C3,RDH12,RBP1,RPE65 |
| Saline | P10-21 | Tight Junction Signaling | 1.41 | 0.102 |  | CLDN10,F2RL2,HSF1,CDK4,MPP5,CTNNA1,SNAP25,PRKCZ,NSF,PPP2R3A,PRKAR1B,STX16,PPP2R2C,STX4,NAPA,CLDN3,TNFRSF11B |
| Saline | P10-21 | Ubiquinol-10 Biosynthesis (Eukaryotic) | 1.35 | 0.161 |  | CYP7B1,MICAL2,CYP26A1,BCKDHA,BCKDHB |

Table S1C.

| *Group* | *Comparison* | *Ingenuity Canonical Pathways* | *-log(p-value)* | *Ratio* | *z-score* | *Molecules* |
| --- | --- | --- | --- | --- | --- | --- |
| VPA | P10-21 | Actin Cytoskeleton Signaling | 3.24 | 0.134 | -1.826 | RAC2,RAF1,F2R,MAPK1,PDGFA,ARHGEF7,PIP4K2B,PPP1CB,LIMK1,PTK2,ROCK2,KL,FLNA,BAIAP2,TMSB10/TMSB4X,MAP2K1,ACTN1,VAV2,ITGB1,TIAM1,CSK,ACTN2,FGD1,GSN,APC,ROCK1,PAK3,APC2,MYH3,ARHGAP35,PIP4K2C |
| VPA | P10-21 | Agrin Interactions at Neuromuscular Junction | 1.57 | 0.143 | -1.414 | ITGB1,PTK2,RAC2,PKLR,MAPK1,PAK3,ARHGEF7,MAPK8,UTRN,AGRN |
| VPA | P10-21 | AMPK Signaling | 1.65 | 0.112 | 1.291 | ADRA2B,CHRNA4,MAPK1,SLC2A1,CPT1B,CHRNB1,SMARCE1,CCND1,PPM1G,SMARCD3,NAD+,PFKM,CCNA2,KL,PPM1B,PPP2R3A,FASN,PRKAA2,ACACA,PPP2R2C,ADRA2C,HMGCR,CHRNA3 |
| VPA | P10-21 | Apoptosis Signaling | 3.28 | 0.18 | -2.5 | CAPN5,RAF1,CAPN6,MAPK1,CASP3,NFKBIE,BIRC6,MAPK8,APAF1,BAK1,CDK1,ROCK1,BCL2L1,CAPN2,MAP2K1,CASP7 |
| VPA | P10-21 | B Cell Receptor Signaling | 1.76 | 0.116 | 0.218 | VAV2,GAB2,RAF1,RAC2,MAP3K9,CAMK4,PTK2B,MAPK1,NFKBIE,EGR1,CSK,MAPK8,FCGR2B,PTK2,BCL2L1,MAP3K10,CAMK2A,KL,PPP3R1,MAP2K1,PPP3CA,CAMK2G |
| VPA | P10-21 | CCR3 Signaling in Eosinophils | 1.5 | 0.119 |  | RAF1,CAMK4,MAPK1,GNB5,PPP1CB,ITPR1,PRKCZ,LIMK1,ROCK2,ROCK1,GNAI3,PLCB4,PAK3,KL,PLCB1,MAP2K1 |
| VPA | P10-21 | CD27 Signaling in Lymphocytes | 1.52 | 0.154 | 0.378 | BCL2L1,MAP3K9,MAP3K10,CASP3,NFKBIE,MAPK8,APAF1,MAP2K1 |
| VPA | P10-21 | Circadian Rhythm Signaling | 1.44 | 0.171 |  | GRIN2A,NR1D1,GRIN2D,BHLHE40,GRINA,GRIN3A |
| VPA | P10-21 | D-myo-inositol (1,4,5)-Trisphosphate Biosynthesis | 1.34 | 0.162 |  | PLCB4,PLCE1,PIP4K2B,PLCB1,PLCH1,PIP4K2C |
| VPA | P10-21 | Ephrin Receptor Signaling | 2.4 | 0.13 | -0.471 | ITGB1,RAC2,RAF1,GRIN2A,NGEF,MAPK1,PDGFA,Wasl,GRIN2D,SH2D3C,GNB5,LIMK1,GRINA,GRIN3A,ROCK1,PTK2,ROCK2,GNAI3,PAK3,EPHA5,EPHB3,EFNB3,MAP2K1 |
| VPA | P10-21 | Fatty Acid β-oxidation I | 2.39 | 0.2 |  | ACSL3,SLC27A5,SLC27A2,ACSL6,ACSL4,SLC27A3,ACAA2,ECI1,NAD+ |
| VPA | P10-21 | Glioblastoma Multiforme Signaling | 1.34 | 0.11 | -1.414 | RAF1,MAPK1,PDGFA,ITPR1,CCND1,APC,MYC,PLCB4,PLCE1,RHOG,RND3,IGF1,KL,PDGFRA,WNT4,PLCB1,MAP2K1,CDK2 |
| VPA | P10-21 | Glioma Signaling | 1.37 | 0.119 | -0.832 | RAF1,CAMK4,MAPK1,PDGFA,SUV39H1,CCND1,PRKCZ,CAMK2A,IGF1,KL,TGFA,PDGFRA,MAP2K1,CAMK2G |
| VPA | P10-21 | Gluconeogenesis I | 1.36 | 0.152 |  | PGAM1,ME3,ME1,MDH1,ALDOC,FBP2,NAD+ |
| VPA | P10-21 | GM-CSF Signaling | 1.43 | 0.135 | 0 | BCL2L1,RAF1,CAMK2A,MAPK1,KL,PPP3R1,CCND1,MAP2K1,PPP3CA,CAMK2G |
| VPA | P10-21 | Gαi Signaling | 2.93 | 0.154 | 0.471 | ADRA2B,RAF1,GPR17,RALA,MAPK1,NPY1R,GNB5,HRH3,GABBR2,GNAI3,LPAR1,ADCY5,S1PR1,RGS14,ADRA2C,HTR1F,ADCY10,ADCY8,HCAR2 |
| VPA | P10-21 | HIPPO signaling | 1.68 | 0.138 | -0.447 | DLG1,PPP1R14C,TEAD1,PPP1R7,PPP2R3A,PPP1CB,PPP1R11,PPP2R2C,PPP1R14B,LATS1,PRKCZ,SKP2 |
| VPA | P10-21 | Integrin Signaling | 1.83 | 0.114 | -0.626 | ITGB1,CAPN5,RAC2,RAF1,CAPN6,RALA,MAPK1,Wasl,ACTN2,ARHGEF7,MAPK8,PPP1CB,GSN,ROCK1,PTK2,RHOG,RND3,PAK3,ARF3,KL,CAPN2,TSPAN6,MAP2K1,Arf2,ACTN1 |
| VPA | P10-21 | LXR/RXR Activation | 1.4 | 0.117 | 0.302 | SCD,HPX,VTN,IL33,ALB,LYZ,IL1RN,FASN,ACACA,HMGCR,IL1RAP,CLU,APOD,RBP4,TNFRSF11B |
| VPA | P10-21 | Mevalonate Pathway I | 1.4 | 0.185 |  | MVD,IDI1,ACAT1,HMGCR,HMGCS1 |
| VPA | P10-21 | Mitochondrial Dysfunction | 1.55 | 0.112 |  | ATP5J,COX17,CASP3,NDUFS7,PRDX5,CPT1B,XDH,MAPK8,LRRK2,UQCR11,GPX7,NDUFA1,NAD+,VPS9D1,NDUFB9,COX6B2,NDUFA6,ATPAF2,CYB5A,ACO1,NDUFA8 |
| VPA | P10-21 | Molecular Mechanisms of Cancer | 3.43 | 0.12 |  | GAB2,RAF1,RAC2,RALA,MAPK1,ARHGEF7,NFKBIE,SUV39H1,CCND1,PRKCZ,PTK2,MYC,CTNNA2,CAMK2A,RHOG,ADCY5,KL,WNT4,PLCB1,ARHGEF3,ADCY8,MAP2K1,CDC25A,ITGB1,CDC25C,STK36,CASP3,PTCH1,APAF1,MAPK8,AURKA,BAK1,APC,BCL2L1,GNAI3,PLCB4,RND3,PAK3,RASGRP1,ADCY10,ARHGEF9,GLI1,NOTCH1,CASP7,CDK2,CAMK2G |
| VPA | P10-21 | Oleate Biosynthesis II (Animals) | 1.37 | 0.211 |  | SCD,FADS2,Ptprt,CYB5A |
| VPA | P10-21 | PAK Signaling | 2.58 | 0.155 | -2 | ITGB1,RAF1,PTK2B,MAPK1,CASP3,PDGFA,ARHGEF7,MAPK8,LIMK1,PTK2,PAK3,KL,PDGFRA,EPHB3,DSCAM,MAP2K1 |
| VPA | P10-21 | Pancreatic Adenocarcinoma Signaling | 1.61 | 0.125 | -1.732 | RAF1,RALA,MAPK1,SUV39H1,MAPK8,HBEGF,CCND1,BIRC5,RAD51,BCL2L1,KL,TGFA,MAP2K1,NOTCH1,CDK2 |
| VPA | P10-21 | Phospholipase C Signaling | 1.33 | 0.102 | 0 | RAF1,RALA,CAMK4,MAPK1,ARHGEF7,GNB5,PPP1CB,FCGR2B,PRKCZ,RHOG,PLCE1,ADCY5,PPP3R1,HDAC11,PLCB1,ARHGEF3,ADCY8,MAP2K1,PPP3CA,ITGB1,ITPR1,PLCB4,RND3,ADCY10,ARHGEF9 |
| VPA | P10-21 | PPARα/RXRα Activation | 1.41 | 0.109 | -1.134 | RAF1,GPD1,MAPK1,NFKBIE,CPT1B,MAPK8,MED12,NR2F1,PLCB4,CHD5,PLCE1,ADCY5,FASN,PRKAA2,PLCB1,ADCY10,ADCY8,IL1RAP,MAP2K1,ACVR1C |
| VPA | P10-21 | Pyridoxal 5'-phosphate Salvage Pathway | 2.68 | 0.176 |  | PDXK,MAP3K9,MAPK1,MAPK8,MAPK6,TTK,CDK1,LIMK1,PRKX,PAK3,PRKAA2,MAP2K1,CDK2 |
| VPA | P10-21 | Rac Signaling | 1.94 | 0.133 | 0.258 | ITGB1,RAF1,TIAM1,PTK2B,MAPK1,MAPK8,PIP4K2B,PRKCZ,LIMK1,PTK2,ARFIP2,PAK3,KL,BAIAP2,MAP2K1,PIP4K2C |
| VPA | P10-21 | RAN Signaling | 1.45 | 0.222 |  | CSE1L,TNPO1,RANBP1,KPNA2 |
| VPA | P10-21 | RANK Signaling in Osteoclasts | 1.52 | 0.127 | 0.577 | MAP3K10,MAP3K9,RAF1,CAMK4,PTK2B,MAPK1,KL,PPP3R1,NFKBIE,MAPK8,GSN,MAP2K1,PPP3CA |
| VPA | P10-21 | Regulation of Actin-based Motility by Rho | 1.83 | 0.14 | -1.155 | ITGB1,ROCK1,RAC2,RHOG,RND3,PAK3,Wasl,BAIAP2,PIP4K2B,PPP1CB,GSN,PIP4K2C,LIMK1 |
| VPA | P10-21 | RhoA Signaling | 2.89 | 0.153 | 0.471 | NGEF,PTK2B,SEPT9,SEPT3,RTKN,PPP1CB,PIP4K2B,LIMK1,ROCK2,PTK2,ROCK1,IGF1,LPAR1,LPAR2,RND3,BAIAP2,ARHGAP12,ARHGAP35,PIP4K2C |
| VPA | P10-21 | RhoGDI Signaling | 1.76 | 0.117 | 0.943 | ITGB1,ARHGDIG,ARHGEF7,GNB5,PIP4K2B,GRIP1,LIMK1,CDH19,ROCK1,ROCK2,GNAI3,RHOG,RND3,PAK3,CDH8,ARHGAP12,ARHGAP35,ARHGEF3,ARHGEF9,CDH13,PIP4K2C |
| VPA | P10-21 | Role of Macrophages, Fibroblasts and Endothelial Cells in Rheumatoid Arthritis | 1.54 | 0.102 |  | RAF1,SFRP2,CAMK4,MAPK1,PDGFA,NFKBIE,CCND1,PRKCZ,ROCK2,MYC,PLCE1,CAMK2A,WIF1,KL,PPP3R1,TRAF4,PLCB1,WNT4,MAP2K1,IL1RAP,PPP3CA,FCGR3A/FCGR3B,TNFRSF11B,DAAM1,TCF7L1,APC,IL33,ROCK1,PLCB4,IL1RN,APC2,CAMK2G |
| VPA | P10-21 | Salvage Pathways of Pyrimidine Ribonucleotides | 1.6 | 0.127 |  | MAP3K9,MAPK1,UPP1,MAPK8,MAPK6,TTK,CDK1,LIMK1,NME3,PRKX,PAK3,PRKAA2,MAP2K1,CDK2 |
| VPA | P10-21 | Small Cell Lung Cancer Signaling | 1.34 | 0.126 |  | PTK2,MYC,BCL2L1,KL,NFKBIE,SUV39H1,TRAF4,APAF1,CCND1,CDK2,SKP2 |
| VPA | P10-21 | Sonic Hedgehog Signaling | 2.27 | 0.226 | -1.342 | STK36,GLIS2,PTCH1,HHIP,GLI1,CDK1,CCNB1 |
| VPA | P10-21 | Stearate Biosynthesis I (Animals) | 2.67 | 0.204 |  | ACSL3,SLC27A5,DHCR24,SLC27A2,FASN,ACSL6,ACSL4,SLC27A3,ELOVL1,ACOT7 |
| VPA | P10-21 | γ-linolenate Biosynthesis II (Animals) | 3.76 | 0.333 |  | ACSL3,SLC27A5,SLC27A2,ACSL6,FADS2,ACSL4,SLC27A3,CYB5A |

Table S2. Canonical RNA Sequencing Pathways differing between Saline and VPA amygdala at (A) P10 and (B) P21. Ratio represents the number of genes altered between treatments within the total number of genes in that pathway, and the p-value is from a right-tailed Fisher exact test of this ratio. The z-score represents the predicted change in gene regulation of that pathway.

A.

| *Age* | *Comparison* | *Ingenuity Canonical Pathways* | *-log(p-value)* | *Ratio* | *z-score* | *Molecules* |
| --- | --- | --- | --- | --- | --- | --- |
| P10 | VPA/Saline | Reelin Signaling in Neurons | 3.57 | 0.0978 |  | MAP3K9,APOE,MAP3K10,MAP2K7,MAP3K11,MAPT,HCK,MAPK8IP1,ITGAL |
| P10 | VPA/Saline | Notch Signaling | 2.77 | 0.132 | 2 | NOTCH2,FURIN,NOTCH3,HES5,NOTCH1 |
| P10 | VPA/Saline | SAPK/JNK Signaling | 2.53 | 0.0762 | 1.134 | MAP3K9,MAP3K10,MAP2K7,MAP3K11,NFATC3,DUSP10,MAPK8IP1,MAP4K4 |
| P10 | VPA/Saline | Signaling by Rho Family GTPases | 2.28 | 0.0522 | 0.832 | MAP3K9,MAP2K7,MAP3K11,PTK2B,CDC42EP5,PIP4K2B,DES,CDH19,LIMK1,MAP3K10,WIPF1,BAIAP2,FNBP1 |
| P10 | VPA/Saline | LXR/RXR Activation | 2.02 | 0.0625 | 0 | APOE,IL1A,FASN,APOA5,ABCG1,NCOR2,RXRB,IL1RAP |
| P10 | VPA/Saline | B Cell Receptor Signaling | 1.9 | 0.0526 | 1.897 | MAP3K9,MAP3K10,MAP2K7,MAP3K11,APBB1IP,PTK2B,NFATC3,ABL1,INPPL1,CAMK2G |
| P10 | VPA/Saline | tRNA Splicing | 1.74 | 0.0909 |  | PDE3B,PDE4A,PDE8B,NAD+ |
| P10 | VPA/Saline | Regulation of Actin-based Motility by Rho | 1.69 | 0.0645 | 1.633 | WIPF1,PFN1,BAIAP2,PIP4K2B,FNBP1,LIMK1 |
| P10 | VPA/Saline | PPAR Signaling | 1.69 | 0.0645 | -1.633 | IL1A,PDGFRA,NCOR2,INSR,IL1RAP,MAP4K4 |
| P10 | VPA/Saline | Rac Signaling | 1.68 | 0.0583 | 1.134 | MCF2L,MAP2K7,MAP3K11,PTK2B,BAIAP2,PIP4K2B,LIMK1 |
| P10 | VPA/Saline | IL-15 Production | 1.63 | 0.111 |  | TWF1,MAP3K11,PTK2B |
| P10 | VPA/Saline | Protein Kinase A Signaling | 1.61 | 0.0398 | -0.535 | PTK2B,PTPN23,NFATC3,PDIA3,PTCH1,PDE4A,DUSP15,ANAPC7,ANAPC13,PTPRF,PDE3B,FLNC,DUSP10,PDE8B,CDC16,CAMK2G |
| P10 | VPA/Saline | Fcγ Receptor-mediated Phagocytosis in Macrophages and Monocytes | 1.57 | 0.0606 | 0.816 | PLA2G6,NCF1,PTK2B,GPLD1,HCK,TLN1 |
| P10 | VPA/Saline | STAT3 Pathway | 1.56 | 0.0676 | 2.236 | MAP3K9,MAP3K10,MAP3K11,PDGFRA,INSR |
| P10 | VPA/Saline | G-Protein Coupled Receptor Signaling | 1.56 | 0.0432 |  | ADRA2B,GPR17,PTK2B,PDE3B,NPY1R,GRM1,PDE4A,PDE8B,RAPGEF3,RPS6KA1,CRHR1,CAMK2G |
| P10 | VPA/Saline | CD27 Signaling in Lymphocytes | 1.5 | 0.0769 | 2 | MAP3K9,MAP3K10,MAP2K7,MAP3K11 |
| P10 | VPA/Saline | Semaphorin Signaling in Neurons | 1.48 | 0.0755 |  | SEMA4D,DPYSL5,FNBP1,LIMK1 |
| P10 | VPA/Saline | TR/RXR Activation | 1.47 | 0.0571 |  | UCP2,PDE3B,FASN,APOA5,NCOR2,RXRB |
| P10 | VPA/Saline | Axonal Guidance Signaling | 1.46 | 0.0374 |  | MMP21,PAPPA,PFN1,RGS3,SLIT1,NFATC3,PDIA3,SEMA5A,PTCH1,ABL1,VEGFB,DPYSL5,LIMK1,WIPF1,SEMA6D,SEMA4D,BAIAP2 |
| P10 | VPA/Saline | cAMP-mediated signaling | 1.45 | 0.0444 | 1.265 | ADRA2B,GPR17,PDE3B,NPY1R,PDE4A,PDE8B,RAPGEF3,RPS6KA1,CRHR1,CAMK2G |
| P10 | VPA/Saline | Galactose Degradation I (Leloir Pathway) | 1.4 | 0.143 |  | GALT,GALM |
| P10 | VPA/Saline | Cdc42 Signaling | 1.38 | 0.0473 | -0.447 | WIPF1,MAP3K11,LLGL1,CDC42EP5,BAIAP2,EXOC5,HLA-DOB,LIMK1 |
| P10 | VPA/Saline | NAD biosynthesis II (from tryptophan) | 1.37 | 0.0882 |  | AFMID,ABL1,NAD+ |
| P10 | VPA/Saline | Ethanol Degradation IV | 1.31 | 0.0833 |  | ALDH1A1,CYGB,NAD+ |

B.

| *Age* | *Comparison* | *Ingenuity Canonical Pathways* | *-log(p-value)* | *Ratio* | *z-score* | *Molecules* |
| --- | --- | --- | --- | --- | --- | --- |
| P21 | VPA/Saline | Cholecystokinin/Gastrin-mediated Signaling | 4.04 | 0.0849 |  | RND2,ROCK2,IL1RN,MAPK8,SST,PRKCH,CCK,PTGS2,CCKBR |
| P21 | VPA/Saline | Axonal Guidance Signaling | 3.87 | 0.0441 |  | EPHA7,SLIT1,FZD3,NFATC3,CXCR4,WNT9A,ARHGEF15,SEMA5A,GNA14,ADAMTS2,HERC2,ROCK2,TUBA8,ABLIM3,EPHA5,FIGF,PRKCH,SEMA3C,RTN4R,MYL3 |
| P21 | VPA/Saline | Calcium Signaling | 3.4 | 0.0591 | -1 | HDAC9,GRIN2A,CHRNA4,MYH8,NFATC3,TRPV6,RYR2,MYH3,Tpm1,TRPC3,MYL3 |
| P21 | VPA/Saline | Protein Kinase A Signaling | 3.18 | 0.0423 | 0.775 | TCF4,PTPRG,NFATC3,DUSP6,RYR2,PTPN18,PDE1C,ROCK2,PTPN4,PPP1R3D,PTPRJ,TGFB3,PRKCH,PTGS2,CDC16,NFKBIB,MYL3 |
| P21 | VPA/Saline | Role of Osteoblasts, Osteoclasts and Chondrocytes in Rheumatoid Arthritis | 3.04 | 0.0504 |  | TCF4,MMP3,DKK3,NFATC3,IL1RN,FZD3,WNT9A,MAPK8,CALCR,IL1R1,NFKBIB,XIAP |
| P21 | VPA/Saline | Molecular Mechanisms of Cancer | 2.98 | 0.0419 |  | RND2,TCF4,FZD3,ARHGEF15,WNT9A,MAPK8,GNA14,XIAP,MYC,BCL2L1,CDH1,NLK,APH1A,TGFB3,PRKCH,NFKBIB |
| P21 | VPA/Saline | Role of Macrophages, Fibroblasts and Endothelial Cells in Rheumatoid Arthritis | 2.91 | 0.0444 |  | ROCK2,MYC,TCF4,NLK,MMP3,IL1RN,DKK3,FZD3,NFATC3,WNT9A,FIGF,PRKCH,IL1R1,NFKBIB |
| P21 | VPA/Saline | Colorectal Cancer Metastasis Signaling | 2.82 | 0.0476 | 0 | RND2,MYC,BCL2L1,CDH1,TCF4,MMP3,FZD3,WNT9A,MAPK8,TGFB3,FIGF,PTGS2 |
| P21 | VPA/Saline | GPCR-Mediated Integration of Enteroendocrine Signaling Exemplified by an L Cell | 2.59 | 0.075 |  | ADRB1,SST,CCK,GNA14,VIP,ADCYAP1 |
| P21 | VPA/Saline | Induction of Apoptosis by HIV1 | 2.43 | 0.0833 |  | BCL2L1,CXCR4,MAPK8,NFKBIB,XIAP |
| P21 | VPA/Saline | Amyotrophic Lateral Sclerosis Signaling | 2.32 | 0.0583 |  | PRPH,BCL2L1,GRIN2A,NEFL,FIGF,SOD1,XIAP |
| P21 | VPA/Saline | Polyamine Regulation in Colon Cancer | 2.16 | 0.13 |  | MYC,PSMA8,TCF4 |
| P21 | VPA/Saline | ILK Signaling | 2.12 | 0.0455 | -1.414 | RND2,MYC,CDH1,MYH8,MAPK8,MYH3,FIGF,PTGS2,MYL3 |
| P21 | VPA/Saline | Gα12/13 Signaling | 2.11 | 0.0534 | -0.816 | ROCK2,CDH1,F2RL2,CDH8,MAPK8,NFKBIB,MYL3 |
| P21 | VPA/Saline | IL-8 Signaling | 2.08 | 0.0448 | -0.378 | RND2,ROCK2,BCL2L1,CDH1,MAPK8,FIGF,PRKCH,PTGS2,NFKBIB |
| P21 | VPA/Saline | Gαq Signaling | 2.05 | 0.0476 | 1.134 | RND2,ROCK2,NFATC3,CALCR,PRKCH,GNA14,NFKBIB,AVPR1A |
| P21 | VPA/Saline | Wnt/β-catenin Signaling | 2.02 | 0.0471 | 1.134 | MYC,CDH1,TCF4,NLK,DKK3,FZD3,WNT9A,TGFB3 |
| P21 | VPA/Saline | Signaling by Rho Family GTPases | 1.94 | 0.0402 | -2.333 | RND2,ROCK2,MAP3K9,CDH1,ARHGEF15,CDH8,MAPK8,GNA14,SEPT11,MYL3 |
| P21 | VPA/Saline | Ephrin Receptor Signaling | 1.92 | 0.0452 | -2.236 | ROCK2,EPHA7,GRIN2A,CXCR4,ARHGEF15,EPHA5,FIGF,GNA14 |
| P21 | VPA/Saline | Embryonic Stem Cell Differentiation into Cardiac Lineages | 1.92 | 0.2 |  | T,MESP1 |
| P21 | VPA/Saline | CD27 Signaling in Lymphocytes | 1.91 | 0.0769 |  | BCL2L1,MAP3K9,MAPK8,NFKBIB |
| P21 | VPA/Saline | TNFR2 Signaling | 1.88 | 0.103 |  | MAPK8,NFKBIB,XIAP |
| P21 | VPA/Saline | NRF2-mediated Oxidative Stress Response | 1.72 | 0.0415 |  | ERP29,MGST2,DNAJC6,MAPK8,DNAJA3,PRKCH,SOD1,MGST3 |
| P21 | VPA/Saline | Circadian Rhythm Signaling | 1.66 | 0.0857 |  | GRIN2A,VIP,ADCYAP1 |
| P21 | VPA/Saline | G-Protein Coupled Receptor Signaling | 1.64 | 0.036 |  | GRM2,ADRB1,DUSP6,CNR1,CALCR,GNA14,RGS12,NFKBIB,AVPR1A,PDE1C |
| P21 | VPA/Saline | PCP pathway | 1.63 | 0.0635 | -1 | ROCK2,FZD3,WNT9A,MAPK8 |
| P21 | VPA/Saline | Glutamate Receptor Signaling | 1.61 | 0.0625 |  | GRM2,GRIN2A,SLC1A6,GLS |
| P21 | VPA/Saline | Tight Junction Signaling | 1.58 | 0.0419 |  | MPDZ,F2RL2,YKT6,MYH8,MYH3,TGFB3,MYL3 |
| P21 | VPA/Saline | April Mediated Signaling | 1.56 | 0.0789 |  | NFATC3,MAPK8,NFKBIB |
| P21 | VPA/Saline | Huntington's Disease Signaling | 1.55 | 0.0364 | 0 | PSMA8,BCL2L1,HDAC9,YKT6,GLS,MAPK8,PRKCH,GNA14,STX1A |
| P21 | VPA/Saline | CXCR4 Signaling | 1.51 | 0.0405 | -0.816 | RND2,ROCK2,CXCR4,MAPK8,PRKCH,GNA14,MYL3 |
| P21 | VPA/Saline | B Cell Activating Factor Signaling | 1.5 | 0.075 |  | NFATC3,MAPK8,NFKBIB |
| P21 | VPA/Saline | Hepatic Cholestasis | 1.5 | 0.0402 |  | CYP27A1,IL1RN,MAPK8,TGFB3,PRKCH,IL1R1,NFKBIB |
| P21 | VPA/Saline | Thyroid Cancer Signaling | 1.48 | 0.0732 |  | MYC,CDH1,TCF4 |
| P21 | VPA/Saline | TR/RXR Activation | 1.45 | 0.0476 |  | ENO1,ADRB1,AKR1C3,ME1,THRB |
| P21 | VPA/Saline | IL-10 Signaling | 1.45 | 0.0556 |  | IL1RN,MAPK8,IL1R1,NFKBIB |
| P21 | VPA/Saline | RhoGDI Signaling | 1.44 | 0.0391 | 1.633 | RND2,ROCK2,CDH1,ARHGEF15,CDH8,GNA14,MYL3 |
| P21 | VPA/Saline | Mouse Embryonic Stem Cell Pluripotency | 1.44 | 0.0472 | 0.447 | MYC,TCF4,T,FZD3,XIAP |
| P21 | VPA/Saline | GABA Receptor Signaling | 1.43 | 0.0548 |  | GAD2,GABRG1,KCNQ3,AP2S1 |
| P21 | VPA/Saline | Pyridoxal 5'-phosphate Salvage Pathway | 1.41 | 0.0541 |  | MAP3K9,MAPK8,PLK1,PRKCH |
| P21 | VPA/Saline | MIF Regulation of Innate Immunity | 1.4 | 0.0682 |  | MAPK8,PTGS2,NFKBIB |
| P21 | VPA/Saline | Netrin Signaling | 1.4 | 0.0682 |  | NFATC3,ABLIM3,RYR2 |
| P21 | VPA/Saline | Aryl Hydrocarbon Receptor Signaling | 1.39 | 0.0411 |  | MYC,MGST2,MAPK8,TGFB3,NFIB,MGST3 |
| P21 | VPA/Saline | Salvage Pathways of Pyrimidine Ribonucleotides | 1.38 | 0.0455 |  | MAP3K9,MAPK8,PLK1,PRKCH,UCK2 |
| P21 | VPA/Saline | LPS/IL-1 Mediated Inhibition of RXR Function | 1.37 | 0.0354 |  | MGST2,SLC27A2,IL1RN,MAPK8,CPT1C,NDST4,IL1R1,MGST3 |
| P21 | VPA/Saline | Epithelial Adherens Junction Signaling | 1.36 | 0.0405 |  | CDH1,TCF4,TUBA8,MYH8,MYH3,MYL3 |
| P21 | VPA/Saline | Hepatic Fibrosis / Hepatic Stellate Cell Activation | 1.36 | 0.0374 |  | MYH8,IGFBP3,MYH3,TGFB3,FIGF,IL1R1,MYL3 |
| P21 | VPA/Saline | Gluconeogenesis I | 1.35 | 0.0652 |  | ENO1,ME1,FBP2 |
| P21 | VPA/Saline | Regulation of the Epithelial-Mesenchymal Transition Pathway | 1.34 | 0.037 |  | CDH1,TCF4,FZD3,APH1A,WNT9A,TGFB3,JAG1 |
| P21 | VPA/Saline | Agranulocyte Adhesion and Diapedesis | 1.33 | 0.0368 |  | MMP3,MYH8,CXCR4,IL1RN,MYH3,IL1R1,MYL3 |
| P21 | VPA/Saline | Mitochondrial L-carnitine Shuttle Pathway | 1.3 | 0.0952 |  | SLC27A2,CPT1C |

Table S3. Canonical Proteomic Pathways differing between Saline and VPA amygdala at P21. Ratio represents the number of genes altered between treatments within the total number of genes in that pathway, and the p-value is from a right-tailed Fisher exact test of this ratio. The z-score represents the predicted change in gene regulation of that pathway.

| *Age* | *Comparison* | *Ingenuity Canonical Pathways* | *-log(p-value)* | *Ratio* | *z-score* | *Molecules* |
| --- | --- | --- | --- | --- | --- | --- |
| P21 | VPA/Saline | Actin Cytoskeleton Signaling | 5.06 | .0345 | -0.378 | FLNA,PIP5K1C,ARPC2,MRAS,RDX,TMSB10/TMSB4X,ARPC4,NCKAP1 |
| P21 | VPA/Saline | Actin Nucleation by ARP-WASP Complex | 2.71 | .0536 |  | ARPC2,MRAS,ARPC4 |
| P21 | VPA/Saline | Adenine and Adenosine Salvage I | 1.41 | .111 |  | PNP |
| P21 | VPA/Saline | Agrin Interactions at Neuromuscular Junction | 1.42 | .0286 |  | LAMC1,MRAS |
| P21 | VPA/Saline | Amyotrophic Lateral Sclerosis Signaling | 2.70 | .0333 |  | GRIN1,CAT,GLUL,SLC1A2 |
| P21 | VPA/Saline | Asparagine Degradation I | 1.66 | .200 |  | ASRGL1 |
| P21 | VPA/Saline | Calcium Signaling | 1.31 | .0161 |  | GRIN1,RYR2,RAP1A |
| P21 | VPA/Saline | Clathrin-mediated Endocytosis Signaling | 3.58 | .0300 |  | AP2A1,PIP5K1C,ARPC2,AP2B1,AP2A2,ARPC4 |
| P21 | VPA/Saline | CTLA4 Signaling in Cytotoxic T Lymphocytes | 2.02 | .0303 |  | AP2A1,AP2B1,AP2A2 |
| P21 | VPA/Saline | Endometrial Cancer Signaling | 1.46 | .0303 |  | MRAS,CTNNB1 |
| P21 | VPA/Saline | Ephrin B Signaling | 1.36 | .0267 |  | MRAS,CTNNB1 |
| P21 | VPA/Saline | Ephrin Receptor Signaling | 2.95 | .0282 | -1.342 | GRIN1,ARPC2,MRAS,RAP1A,ARPC4 |
| P21 | VPA/Saline | Epithelial Adherens Junction Signaling | 4.30 | .0405 |  | CDH2,ARPC2,MRAS,CTNNB1,RAP1A,ARPC4 |
| P21 | VPA/Saline | fMLP Signaling in Neutrophils | 1.72 | .0234 |  | ARPC2,MRAS,ARPC4 |
| P21 | VPA/Saline | GABA Receptor Signaling | 4.75 | .0685 |  | AP2A1,AP2B1,MRAS,GABBR1,AP2A2 |
| P21 | VPA/Saline | Germ Cell-Sertoli Cell Junction Signaling | 1.37 | .0170 |  | CDH2,MRAS,CTNNB1 |
| P21 | VPA/Saline | Glutamate Receptor Signaling | 2.55 | .0469 |  | GRIN1,GLUL,SLC1A2 |
| P21 | VPA/Saline | Glutamine Biosynthesis I | 1.52 | .0143 |  | GLUL |
| P21 | VPA/Saline | Glutathione Biosynthesis | 1.32 | .0909 |  | GSS |
| P21 | VPA/Saline | Glutathione Redox Reactions II | 1.46 | .125 |  | GLRX |
| P21 | VPA/Saline | Glycolysis I | 1.85 | .0488 |  | PFKL,Tpi1 (includes others) |
| P21 | VPA/Saline | Guanine and Guanosine Salvage I | 1.41 | .111 |  | PNP |
| P21 | VPA/Saline | Gα12/13 Signaling | 1.69 | .0229 |  | CDH2,MRAS,CTNNB1 |
| P21 | VPA/Saline | Gαi Signaling | 1.77 | .0244 |  | MRAS,GABBR1,RAP1A |
| P21 | VPA/Saline | Gαs Signaling | 1.86 | .0265 |  | RYR2,MRAS,RAP1A |
| P21 | VPA/Saline | HIF1α Signaling | 1.82 | .0256 |  | MRAS,TCEB1,Ldha/RGD1562690 |
| P21 | VPA/Saline | Integrin Signaling | 1.79 | .0182 | -1 | ARPC2,MRAS,RAP1A,ARPC4 |
| P21 | VPA/Saline | Lipid Antigen Presentation by CD1 | 3.70 | .115 |  | AP2A1,AP2B1,AP2A2 |
| P21 | VPA/Saline | Molecular Mechanisms of Cancer | 1.57 | .0131 |  | RABIF,PA2G4,MRAS,CTNNB1,RAP1A |
| P21 | VPA/Saline | Neuroprotective Role of THOP1 in Alzheimer's Disease | 2.96 | .0652 |  | PDYN,AGT,APP |
| P21 | VPA/Saline | NRF2-mediated Oxidative Stress Response | 1.98 | .0207 |  | CAT,MRAS,CLPP,GSTP1 |
| P21 | VPA/Saline | Ovarian Cancer Signaling | 1.57 | .0205 |  | PA2G4,MRAS,CTNNB1 |
| P21 | VPA/Saline | Prostate Cancer Signaling | 3.03 | .0408 |  | PA2G4,MRAS,CTNNB1,GSTP1 |
| P21 | VPA/Saline | Protein Kinase A Signaling | 1.49 | .0124 | 1 | FLNA,RYR2,CTNNB1,RAP1A,SIRPA |
| P21 | VPA/Saline | Protein Ubiquitination Pathway | 1.56 | .0154 |  | PSMB3,UBE2M,TCEB1,UBE3A |
| P21 | VPA/Saline | Pyruvate Fermentation to Lactate | 1.36 | .100 |  | Ldha/RGD1562690 |
| P21 | VPA/Saline | Rac Signaling | 3.72 | .0417 | -1.342 | PIP5K1C,ARPC2,MRAS,ARPC4,NCKAP1 |
| P21 | VPA/Saline | Regulation of Actin-based Motility by Rho | 2.09 | .0323 |  | PIP5K1C,ARPC2,ARPC4 |
| P21 | VPA/Saline | Remodeling of Epithelial Adherens Junctions | 2.47 | .0441 |  | ARPC2,CTNNB1,ARPC4 |
| P21 | VPA/Saline | Renal Cell Carcinoma Signaling | 2.23 | .0361 |  | MRAS,TCEB1,RAP1A |
| P21 | VPA/Saline | RhoA Signaling | 2.65 | .0323 | 0 | PIP5K1C,ARPC2,RDX,ARPC4 |
| P21 | VPA/Saline | RhoGDI Signaling | 4.84 | .0391 | 1.134 | CDH2,PIP5K1C,ARPC2,MRAS,RDX,ARPC4,ARHGDIB |
| P21 | VPA/Saline | Signaling by Rho Family GTPases | 3.08 | .0241 | -0.816 | CDH2,PIP5K1C,ARPC2,MRAS,RDX,ARPC4 |
| P21 | VPA/Saline | Sulfate Activation for Sulfonation | 1.46 | .125 |  | PAPSS1 |
| P21 | VPA/Saline | Superoxide Radicals Degradation | 1.36 | .100 |  | CAT |
| P21 | VPA/Saline | Synaptic Long Term Potentiation | 1.72 | .0234 |  | GRIN1,MRAS,RAP1A |
| P21 | VPA/Saline | Thyroid Cancer Signaling | 1.85 | .0488 |  | MRAS,CTNNB1 |
| P21 | VPA/Saline | Virus Entry via Endocytic Pathways | 3.93 | .0463 |  | AP2A1,FLNA,AP2B1,MRAS,AP2A2 |
| P21 | VPA/Saline | Xanthine and Xanthosine Salvage | 1.46 | .125 |  | PNP |

Table S4. Diseases and Functions RNA Sequencing Categories differing from P10-21 in (A) both VPA and Saline amygdala, (B) exclusively in Saline amygdala or (C) exclusively in VPA amygdala both VPA and Saline amygdala. The z-score represents the predicted change in gene regulation of that pathway, and the p-value is from a right-tailed Fisher exact test of the ratio of number of genes altered between treatments within the total number of genes in that pathway.

A.

| *Group* | *Comparison* | *Diseases or Functions Annotation* | *p-Value* | *Predicted Activation State* | *Activation z-score* | *# Molecules* |
| --- | --- | --- | --- | --- | --- | --- |
| Saline | P10-21 | cell cycle progression | 3.03E-11 | Decreased | -2.119 | 138 |
| VPA | P10-21 |  | 1.56E-13 | Decreased | -3.542 | 158 |
| Saline | P10-21 | hepatocellular carcinoma | 0.000000818 | Decreased | -2.131 | 104 |
| VPA | P10-21 |  | 0.000000227 | Decreased | -2.48 | 116 |
| Saline | P10-21 | liver carcinoma | 3.51E-09 | Decreased | -2.131 | 501 |
| VPA | P10-21 |  | 2.39E-11 | Decreased | -2.268 | 569 |
| Saline | P10-21 | neurological signs | 4.15E-27 | Increased | 2.18 | 149 |
| VPA | P10-21 |  | 4.89E-29 | Increased | 2.573 | 164 |

B.

| *Group* | *Comparison* | *Diseases or Functions Annotation* | *p-Value* | *Predicted Activation State* | *Activation z-score* | *# Molecules* |
| --- | --- | --- | --- | --- | --- | --- |
| Saline | P10-21 | cellular homeostasis | 9.57E-08 | Increased | 3.372 | 197 |
| Saline | P10-21 | transport of inorganic cation | 0.00000422 | Increased | 2.062 | 51 |
| Saline | P10-21 | transport of metal ion | 0.0000015 | Increased | 2.062 | 49 |
| Saline | P10-21 | transport of molecule | 1.38E-15 | Increased | 2.137 | 237 |
| Saline | P10-21 | size of body | 0.000019 | Increased | 2.577 | 112 |
| Saline | P10-21 | ataxia | 0.00000114 | Decreased | -2.451 | 45 |
| Saline | P10-21 | generalized seizures | 0.00000132 | Decreased | -2.341 | 20 |
| Saline | P10-21 | Movement Disorders | 5.88E-31 | Decreased | -3.056 | 220 |
| Saline | P10-21 | seizure disorder | 1.33E-20 | Decreased | -3.516 | 108 |
| Saline | P10-21 | seizures | 1.16E-21 | Decreased | -3.44 | 96 |
| Saline | P10-21 | tremor | 0.00000218 | Decreased | -2.928 | 28 |
| Saline | P10-21 | skin lesion | 0.000000467 | Decreased | -2.232 | 531 |
| Saline | P10-21 | skin tumor | 0.000000228 | Decreased | -2.571 | 528 |
| Saline | P10-21 | cell death of cancer cells | 5.37E-08 | Increased | 3.363 | 64 |
| Saline | P10-21 | cell death of osteosarcoma cells | 5.12E-09 | Increased | 4.914 | 29 |
| Saline | P10-21 | cell death of tumor cells | 0.000000113 | Increased | 3.01 | 72 |
| Saline | P10-21 | necrosis of tumor | 5.94E-08 | Increased | 3.01 | 74 |
| Saline | P10-21 | metabolism of membrane lipid derivative | 3.5E-11 | Increased | 2.101 | 70 |
| Saline | P10-21 | synthesis of lipid | 0.000000211 | Increased | 2.625 | 99 |
| Saline | P10-21 | glucose metabolism disorder | 0.00000831 | Decreased | -2.024 | 166 |
| Saline | P10-21 | sensation | 0.00000113 | Increased | 2.224 | 51 |

C.

| *Group* | *Comparison* | *Diseases or Functions Annotation* | *p-Value* | *Predicted Activation State* | *Activation z-score* | *# Molecules* |
| --- | --- | --- | --- | --- | --- | --- |
| VPA | P10-21 | morbidity or mortality | 1.73E-23 | Increased | 4.529 | 403 |
| VPA | P10-21 | neonatal death | 0.0000104 | Increased | 3.378 | 69 |
| VPA | P10-21 | organismal death | 2.27E-24 | Increased | 4.336 | 402 |
| VPA | P10-21 | cytokinesis | 0.000000418 | Decreased | -2.052 | 34 |
| VPA | P10-21 | mitosis | 2.7E-10 | Decreased | -2.782 | 80 |
| VPA | P10-21 | branching of cells | 6.88E-13 | Decreased | -2.263 | 83 |
| VPA | P10-21 | formation of plasma membrane projections | 3.08E-21 | Decreased | -2.058 | 138 |
| VPA | P10-21 | generation of cells | 1.69E-19 | Decreased | -2.001 | 332 |
| VPA | P10-21 | proliferation of cells | 1.21E-26 | Decreased | -2.222 | 519 |
| VPA | P10-21 | sprouting | 1.43E-12 | Decreased | -2.341 | 85 |
| VPA | P10-21 | cell movement | 3.46E-17 | Decreased | -3.031 | 311 |
| VPA | P10-21 | cell movement of neurons | 9.12E-11 | Decreased | -3.285 | 55 |
| VPA | P10-21 | invasion of tumor cell lines | 0.00000247 | Decreased | -2.178 | 75 |
| VPA | P10-21 | migration of cells | 1.78E-17 | Decreased | -2.882 | 288 |
| VPA | P10-21 | congenital anomaly of central nervous system | 9.96E-08 | Increased | 3.484 | 59 |
| VPA | P10-21 | congenital encephalopathy | 9.96E-08 | Increased | 3.484 | 59 |
| VPA | P10-21 | congenital malformation of brain | 0.000000178 | Increased | 3.484 | 58 |
| VPA | P10-21 | death of embryo | 0.00000125 | Increased | 2.177 | 31 |
| VPA | P10-21 | midline defect | 2.17E-08 | Increased | 3.434 | 53 |
| VPA | P10-21 | neural tube defect | 0.00000011 | Increased | 3.235 | 33 |
| VPA | P10-21 | dyskinesia | 9.02E-28 | Increased | 2.194 | 154 |
| VPA | P10-21 | neuromuscular disease | 1.92E-27 | Increased | 2.22 | 199 |
| VPA | P10-21 | astrocytoma | 0.000000683 | Decreased | -2.2 | 116 |
| VPA | P10-21 | carcinoma in lung | 1.47E-10 | Decreased | -2.027 | 250 |
| VPA | P10-21 | cell proliferation of tumor cell lines | 2.27E-09 | Decreased | -3.027 | 93 |
| VPA | P10-21 | cell transformation | 0.00000343 | Decreased | -2.325 | 71 |
| VPA | P10-21 | epithelial cancer | 4.21E-43 | Decreased | -3.174 | 1355 |
| VPA | P10-21 | genital tract cancer | 1.85E-15 | Decreased | -2.207 | 623 |
| VPA | P10-21 | glioblastoma cancer | 0.00000235 | Decreased | -2 | 63 |
| VPA | P10-21 | glioma cancer | 0.00000121 | Decreased | -2.186 | 65 |
| VPA | P10-21 | gliomatosis | 2.12E-08 | Decreased | -2.556 | 140 |
| VPA | P10-21 | growth of carcinoma | 0.0000027 | Decreased | -2.265 | 29 |
| VPA | P10-21 | growth of malignant tumor | 2.18E-08 | Decreased | -2.035 | 77 |
| VPA | P10-21 | malignant neoplasm of male genital organ | 1.92E-09 | Decreased | -2.207 | 212 |
| VPA | P10-21 | malignant solid tumor | 2.77E-41 | Decreased | -2.006 | 1413 |
| VPA | P10-21 | mammary tumor | 2.74E-19 | Decreased | -2.105 | 361 |
| VPA | P10-21 | neoplasia of epithelial tissue | 1.63E-44 | Decreased | -3.091 | 1363 |
| VPA | P10-21 | pelvic cancer | 4.59E-15 | Decreased | -2.207 | 655 |
| VPA | P10-21 | prostate cancer | 1.14E-08 | Decreased | -2.207 | 202 |
| VPA | P10-21 | skin cancer | 9.03E-08 | Decreased | -2.535 | 583 |
| VPA | P10-21 | tumor in nervous system | 3.01E-08 | Decreased | -2.114 | 169 |
| VPA | P10-21 | tumorigenesis of tissue | 9.1E-45 | Decreased | -3.131 | 1377 |
| VPA | P10-21 | renal lesion | 3.46E-10 | Increased | 2.182 | 198 |
| VPA | P10-21 | concentration of lipid | 3.97E-08 | Decreased | -2.258 | 126 |
| VPA | P10-21 | axonogenesis | 2.58E-09 | Decreased | -2.055 | 49 |
| VPA | P10-21 | branching of neurons | 6.05E-14 | Decreased | -2.045 | 72 |
| VPA | P10-21 | development of neurons | 4.44E-24 | Decreased | -3.1 | 176 |
| VPA | P10-21 | migration of neurons | 9.8E-10 | Decreased | -3.228 | 52 |
| VPA | P10-21 | neuritogenesis | 3.07E-21 | Decreased | -2.148 | 135 |
| VPA | P10-21 | synaptic transmission of cells | 1.59E-09 | Increased | 2.816 | 34 |
| VPA | P10-21 | synaptic transmission of pyramidal neurons | 6.49E-08 | Increased | 2.16 | 12 |

Table S5. Diseases and Functions RNA Sequencing Categories differing between Saline and VPA amygdala at (A) P10 and (B) P21. The z-score represents the predicted change in gene regulation of that pathway, and the p-value is from a right-tailed Fisher exact test of the ratio of number of genes altered between treatments within the total number of genes in that pathway.

A.

| *Age* | *Comparion* | *Diseases or Functions Annotation* | *p-Value* | *Predicted Activation State* | *Activation z-score* | *# Molecules* |
| --- | --- | --- | --- | --- | --- | --- |
| P10 | VPA/Saline | morbidity or mortality | 5.93E-12 | Decreased | -7.245 | 142 |
| P10 | VPA/Saline | organismal death | 9.34E-12 | Decreased | -7.268 | 140 |
| P10 | VPA/Saline | differentiation of cells | 5.15E-14 | Increased | 3.329 | 137 |
| P10 | VPA/Saline | generation of cells | 6.07E-12 | Increased | 2.764 | 123 |
| P10 | VPA/Saline | microtubule dynamics | 2.4E-09 | Increased | 2.645 | 75 |
| P10 | VPA/Saline | organization of cytoskeleton | 2.75E-09 | Increased | 2.703 | 85 |
| P10 | VPA/Saline | formation of cellular protrusions | 3.48E-09 | Increased | 3.196 | 63 |
| P10 | VPA/Saline | proliferation of cells | 7.67E-09 | Increased | 2.065 | 165 |
| P10 | VPA/Saline | formation of plasma membrane projections | 1.23E-08 | Increased | 2.796 | 47 |
| P10 | VPA/Saline | quantity of cells | 0.00000493 | Increased | 4.374 | 91 |
| P10 | VPA/Saline | sprouting | 0.00000601 | Increased | 2.505 | 30 |
| P10 | VPA/Saline | endocytosis | 0.00000665 | Increased | 2.041 | 32 |
| P10 | VPA/Saline | cell survival | 0.0000119 | Increased | 3.438 | 58 |
| P10 | VPA/Saline | branching of cells | 0.000047 | Increased | 2.939 | 27 |
| P10 | VPA/Saline | differentiation of central nervous system cells | 0.0000681 | Increased | 2.121 | 12 |
| P10 | VPA/Saline | cell viability | 0.000154 | Increased | 3.153 | 49 |
| P10 | VPA/Saline | differentiation of embryonic tissue | 0.000749 | Increased | 2.169 | 17 |
| P10 | VPA/Saline | quantity of adipose tissue | 0.000756 | Increased | 2.249 | 18 |
| P10 | VPA/Saline | proliferation of blood cells | 0.00106 | Increased | 2.187 | 44 |
| P10 | VPA/Saline | colony formation of cells | 0.00115 | Increased | 2.777 | 22 |
| P10 | VPA/Saline | development of body trunk | 5.35E-09 | Increased | 2.409 | 78 |
| P10 | VPA/Saline | mass of organism | 0.00000185 | Increased | 2.635 | 27 |
| P10 | VPA/Saline | size of body | 0.0000136 | Increased | 4.247 | 51 |
| P10 | VPA/Saline | formation of muscle | 0.000112 | Increased | 2 | 26 |
| P10 | VPA/Saline | phosphorylation of protein | 0.000101 | Increased | 2.154 | 39 |
| P10 | VPA/Saline | organization of cytoplasm | 3.48E-09 | Increased | 2.703 | 91 |
| P10 | VPA/Saline | cell movement | 2.33E-12 | Increased | 2.878 | 120 |
| P10 | VPA/Saline | migration of cells | 9.5E-11 | Increased | 2.916 | 107 |
| P10 | VPA/Saline | transport of molecule | 0.000047 | Increased | 2.977 | 80 |
| P10 | VPA/Saline | urination disorder | 0.000884 | Decreased | -2.028 | 19 |
| P10 | VPA/Saline | Growth Failure | 0.00000628 | Decreased | -4.626 | 40 |
| P10 | VPA/Saline | Hypoplasia | 0.000399 | Decreased | -4.139 | 30 |
| P10 | VPA/Saline | hypoplasia of organ | 0.000456 | Decreased | -3.862 | 27 |
| P10 | VPA/Saline | dysgenesis | 0.000727 | Decreased | -4.14 | 31 |
| P10 | VPA/Saline | tremor | 0.0000311 | Decreased | -3.274 | 14 |
| P10 | VPA/Saline | Movement Disorders | 0.000151 | Decreased | -4.372 | 58 |
| P10 | VPA/Saline | hyperactive behavior | 0.000142 | Decreased | -2.902 | 13 |
| P10 | VPA/Saline | cancer | 1.75E-16 | Increased | 2.981 | 464 |
| P10 | VPA/Saline | malignant solid tumor | 1.27E-15 | Increased | 2.129 | 458 |
| P10 | VPA/Saline | tumorigenesis of tissue | 7.3E-15 | Increased | 2.019 | 442 |
| P10 | VPA/Saline | transformation of fibroblast cell lines | 0.000301 | Increased | 2.813 | 18 |
| P10 | VPA/Saline | cell transformation | 0.000779 | Increased | 2.162 | 26 |
| P10 | VPA/Saline | development of neurons | 2.26E-09 | Increased | 3.019 | 59 |
| P10 | VPA/Saline | neuritogenesis | 3.46E-08 | Increased | 2.796 | 45 |
| P10 | VPA/Saline | axonogenesis | 0.00000643 | Increased | 2.578 | 20 |
| P10 | VPA/Saline | differentiation of neuroglia | 0.000229 | Increased | 2.057 | 14 |
| P10 | VPA/Saline | quantity of neurons | 0.000369 | Increased | 2.766 | 26 |

B.

| *Age* | *Comparison* | *Diseases or Functions Annotation* | *p-Value* | *Predicted Activation State* | *Activation z-score* | *# Molecules* |
| --- | --- | --- | --- | --- | --- | --- |
| P21 | VPA/Saline | neuronal cell death | 3.21E-08 | Decreased | -2.095 | 41 |
| P21 | VPA/Saline | cell death of sympathetic neuron | 0.00138 | Decreased | -2.217 | 5 |
| P21 | VPA/Saline | morbidity or mortality | 0.00000278 | Increased | 5.105 | 96 |
| P21 | VPA/Saline | organismal death | 0.00000279 | Increased | 5.186 | 95 |
| P21 | VPA/Saline | condensation of cells | 0.000173 | Decreased | -2 | 4 |
| P21 | VPA/Saline | mineralization of cells | 0.0014 | Decreased | -2 | 6 |
| P21 | VPA/Saline | size of body | 0.000751 | Decreased | -3.028 | 36 |
| P21 | VPA/Saline | ion homeostasis of cells | 0.000244 | Decreased | -2.591 | 27 |
| P21 | VPA/Saline | flux of inorganic cation | 0.00126 | Decreased | -2.246 | 15 |
| P21 | VPA/Saline | secretion of molecule | 0.00000129 | Decreased | -2.012 | 32 |
| P21 | VPA/Saline | Growth Failure | 0.000997 | Increased | 2.383 | 27 |
| P21 | VPA/Saline | Infarction | 0.00015 | Decreased | -2.449 | 20 |
| P21 | VPA/Saline | acute coronary syndrome | 0.000379 | Decreased | -2 | 15 |
| P21 | VPA/Saline | infarction of heart | 0.00147 | Decreased | -2.236 | 13 |
| P21 | VPA/Saline | Gastrointestinal Tract Cancer and Tumors | 4.24E-16 | Increased | 2.472 | 291 |
| P21 | VPA/Saline | malignant neoplasm of large intestine | 7.06E-15 | Increased | 2 | 278 |
| P21 | VPA/Saline | colorectal cancer | 8.2E-11 | Increased | 2 | 204 |
| P21 | VPA/Saline | Bleeding | 0.000781 | Increased | 2.211 | 21 |
| P21 | VPA/Saline | memory | 0.000636 | Increased | 2.398 | 15 |

Table S6. Diseases and Functions Proteomic Categories differing between Saline and VPA amygdala at P21.

The z-score represents the predicted change in gene regulation of that pathway, and the p-value is from a right-tailed Fisher exact test of the ratio of number of genes altered between treatments within the total number of genes in that pathway.

| *Age* | *Comparison* | *Diseases or Functions Annotation* | *p-Value* | *Predicted Activation State* | *Activation z-score* | *# Molecules* |
| --- | --- | --- | --- | --- | --- | --- |
| P21 | VPA/Saline | formation of cellular protrusions | 0.000106 | Decreased | -2.453 | 16 |
| P21 | VPA/Saline | migration of fibrosarcoma cell lines | 3.36E-08 | Decreased | -2.4 | 6 |
| P21 | VPA/Saline | proliferation of cells | 0.000898 | Decreased | -2.239 | 35 |
| P21 | VPA/Saline | neuritogenesis | 0.0000494 | Decreased | -2.581 | 13 |
| P21 | VPA/Saline | development of neurons | 0.000108 | Decreased | -2.282 | 15 |
