# Supplementary figures and images for: Developmental disruption of amygdala transcriptome and socioemotional behavior in rats exposed to valproic acid prenatally
Source: Mol Autism. 2017 Aug 1;8:42. doi: 10.1186/s13229-017-0160-x (PMC5539636; doi:10.1186/s13229-017-0160-x)

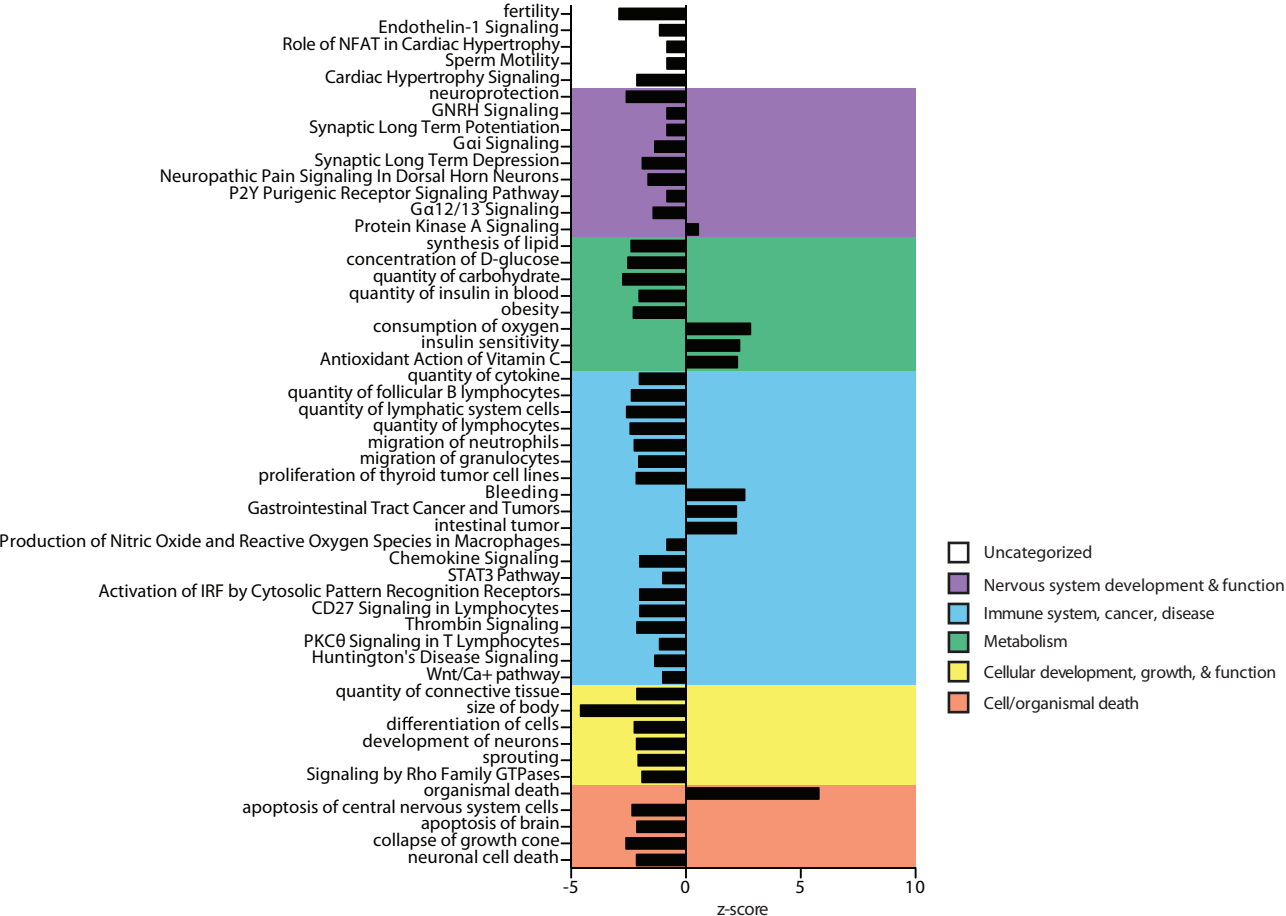

Supplement: Supplementary file 2 — RNA sequencing pathways differentially altered across development between VPA and saline amygdala. Pathways with significant time (P10–21) by treatment (VPA/saline) effects are displayed. Treatment effects did not reach statistical significance after FDR multiple comparison correction, thus Ingenuity Pathway Analyses were run on genes with uncorrected p < 0.05 treatment effects at P10 (n = 542) and P21 (n = 406). Canonical pathways and diseases and functions categories with predicted activation or inhibition differences were broadly categorized into the following groups: cellular development and growth; nervous system development and function; immune system, cancer, disease; cell/organismal death; metabolism; and developmental, neurological, or psychological disorder. (PDF 59 kb) [file 13229_2017_160_MOESM2_ESM.pdf]
